# Inducibility, but not stability, of atrial fibrillation is increased by NOX2 overexpression in mice

**DOI:** 10.1093/cvr/cvab019

**Published:** 2021-01-23

**Authors:** Alexandra S Mighiu, Alice Recalde, Klemen Ziberna, Ricardo Carnicer, Jakub Tomek, Gil Bub, Alison C Brewer, Sander Verheule, Ajay M Shah, Jillian N Simon, Barbara Casadei

**Affiliations:** 1 Division of Cardiovascular Medicine, University of Oxford, L6, West Wing, Oxford OX3 9DU, UK; 2 Department of Physiology, Anatomy and Genetics, University of Oxford, Oxford, UK; 3 School of Cardiovascular Medicine & Sciences, King’s College London, British Heart Foundation Centre of Excellence, London, UK; 4 Department of Physiology, Maastricht University, Maastricht, The Netherlands

**Keywords:** NADPH oxidases, Oxidant stress, Arrhythmia (mechanisms), Atorvastatin, Atrial fibrillation

## Abstract

**Aims:**

Gp91-containing NADPH oxidases (NOX2) are a significant source of myocardial superoxide production. An increase in NOX2 activity accompanies atrial fibrillation (AF) induction and electrical remodelling in animal models and predicts incident AF in humans; however, a direct causal role for NOX2 in AF has not been demonstrated. Accordingly, we investigated whether myocardial NOX2 overexpression in mice (NOX2-Tg) is sufficient to generate a favourable substrate for AF and further assessed the effects of atorvastatin, an inhibitor of NOX2, on atrial superoxide production and AF susceptibility.

**Methods and results:**

NOX2-Tg mice showed a 2- to 2.5-fold higher atrial protein content of NOX2 compared with wild-type (WT) controls, which was associated with a significant (twofold) increase in NADPH-stimulated superoxide production (2-hydroxyethidium by HPLC) in left and right atrial tissue homogenates (*P* = 0.004 and *P* = 0.019, respectively). AF susceptibility assessed *in vivo* by transoesophageal atrial burst stimulation was modestly increased in NOX2-Tg compared with WT (probability of AF induction: 88% vs. 69%, respectively; *P* = 0.037), in the absence of significant alterations in AF duration, surface ECG parameters, and LV mass or function. Mechanistic studies did not support a role for NOX2 in promoting electrical or structural remodelling, as high-resolution optical mapping of atrial tissues showed no differences in action potential duration and conduction velocity between genotypes. In addition, we did not observe any genotype difference in markers of fibrosis and inflammation, including atrial collagen content and *Col1a1, Il-1β, Il-6,* and *Mcp-1* mRNA. Similarly, NOX2 overexpression did not have consistent effects on RyR2 Ca^2+^ leak nor did it affect PKA or CaMKII-mediated RyR2 phosphorylation. Finally, treatment with atorvastatin significantly inhibited atrial superoxide production in NOX2-Tg but had no effect on AF induction in either genotype.

**Conclusion:**

Together, these data indicate that while atrial NOX2 overexpression may contribute to atrial arrhythmogenesis, NOX2-derived superoxide production does not affect the electrical and structural properties of the atrial myocardium.

## Graphical Abstract

**Figure:**
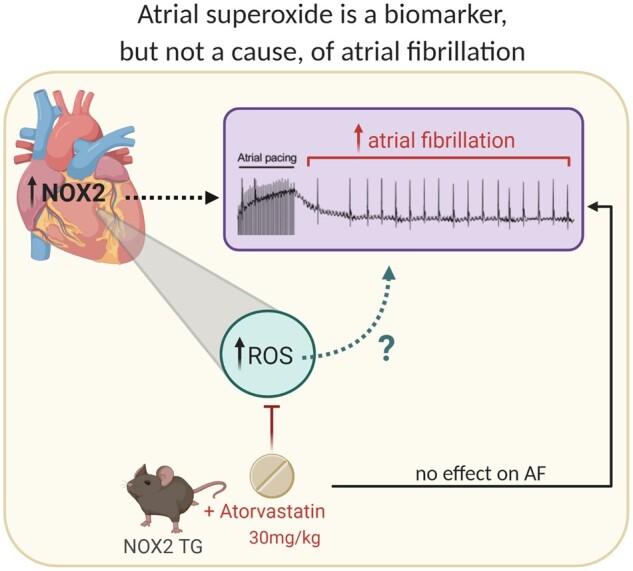

## 1. Introduction

Atrial fibrillation (AF) is the most common sustained arrhythmia, mostly occurring in association with aging and cardiovascular risk factors, and leads to thromboembolic complications and premature death.^1^ Despite an increased understanding of the atrial electrical and structural changes that promote AF self-perpetuation, therapeutic attempts to modify the atrial substrate have shown limited efficacy.^2^^,^^3^

Experimental and clinical evidence implicates oxidative stress in triggering the new onset of AF and promoting AF-related electrical and structural remodelling of the atrial myocardium.^4–12^ In human atrial myocytes, superoxide is predominantly generated by gp91^phox^-containing NADPH oxidases (NOX2),^7^ whose activity is increased in patients with paroxysmal AF and in goats after two weeks of pacing-induced AF.^10^^,^^13^ Moreover, NOX2-dependent atrial superoxide production at the time of cardiac surgery is predictive of AF development in the post-operative period.^14^^,^^15^ Interestingly, perioperative statin therapy, which inhibits NOX2-dependent myocardial superoxide formation,^15^ has been reported to halve the new onset of AF following cardiac surgery in a meta-analysis of small clinical trials (of 40–200 patients).^16^ However, data from the more recent and much larger statin therapy in cardiac surgery (STICS) randomized controlled trial showed no significant effect on the incidence of post-operative AF (odds ratio: 1.04, 95% CI = 0.84–1.34) in 1922 patients randomized to rosuvastatin or placebo,^16^ raising the question of whether NOX2-derived superoxide production is merely a biomarker of increased AF risk or causal factor in the new onset of AF after cardiac surgery. Despite the positive accounts implicating NOX2-derived superoxide in AF, no studies thus far have assessed whether augmenting myocardial NOX2 activity is sufficient to promote AF induction. Here, we investigated whether myocardial-specific overexpression of the human *NOX2* gene generates a substrate for AF in mice and evaluated the efficacy of atorvastatin (ATV) administration for inhibiting atrial NOX2 activity and reducing AF induction.

## 2. Methods

### 2.1. Animal model

Transgenic mice expressing the human *NOX2* gene downstream of the murine myosin light chain-2 (MLC-2v) promoter^17^ were housed in a specific pathogen-free environment. All experiments were performed on male and female adult heterozygous NOX2-Tg mice and their wild-type (WT) littermate controls aged 16 to 20 weeks unless otherwise specified. All animal studies were approved by the local regulatory authority and conducted in accordance with the guidelines from the UK Home Office Guidance on the Operation of Animals (Scientific Procedures) Act 1986 and Directive 2010/63/EU. In all instances, mice were euthanized by cervical dislocation prior to downstream processing.

### 2.2. Treatment

ATV or placebo tablets were ground, mixed with powder rodent chow, and compressed into food pellets. Based on a daily food intake of approximately 3 g chow/mouse, 250 mg ATV was added per kilogram of chow to achieve a dose of 30 mg/kg/day. WT and NOX2-Tg mice received either ATV or placebo for 14 days starting at 14–18 weeks of age. Food consumption and body weight were monitored throughout the dosing period.

### 2.3. RNA isolation and quantitative reverse transcription PCR

Quantitative real-time PCR was performed using TaqMan gene expression assays (ThermoFisher Scientific) for murine *Nox2* (Mm01287743_m1), human *NOX2* (Hs00166163_m1), murine collagen 1A1 (*Col1a1;* Mm00483387_m1), murine monocyte chemoattractant protein-1 (*Mcp-1;* Mm00441242), murine interleukin 1-β (*Il-1β*; Mm00434228_m1), murine (*Il-6;* Mm00446190_m1), with α-actinin (Mm00473657_m1) and GAPDH (Mm99999915_g1) as housekeeping genes, as described in detail in Supplementary material online.

### 2.4. Western blotting

Right atrial (RA) and left atrial (LA) tissue homogenates were probed with the following antibodies: gp91^phox^ (BD Transduction); total and phosphorylated RyR2 at serine-2808 and serine-2814 (ThermoFisher Scientific); α-smooth muscle actin (α-SMA; Cell Signalling); vimentin (Exbio); HRP-conjugated GAPDH (Sigma Aldrich); and total CaMKII (Cell Signalling) and an antiserum to oxidized CaMKII that was kindly provided by Dr ME Anderson, Johns Hopkins University, USA. Detection of primary antibodies was performed using peroxidase-conjugated anti-rabbit or anti-mouse antibodies (both Promega, USA). GAPDH was used as a loading control except for phosphorylated protein levels which were normalized by the respective total protein levels. All western blots were run under reducing conditions apart from those to detect oxidized CaMKII, which was performed under non-reducing conditions to preserve the redox modification. For the detection of RA and LA NOX2, atrial tissues were pooled from two different animals prior to homogenization.

### 2.5. Assays

Atrial superoxide production was measured as the tiron-inhibitable fraction of 2-hydroxyethidium (2-OHE) by HPLC as reported previously^10^ and described in detail in Supplementary material online.

Collagen content was assessed using the Quickzyme Total Collagen Assay kit according to the manufacturer’s instructions. Myocardial and plasma cholesterol was measured using the Amplex Red Cholesterol Assay kit according to manufacturer’s instructions (ThermoFisher Scientific).

### 2.6. Echocardiography

Echocardiography was performed in anaesthetized mice (2% isoflurane) using the VisualSonics Vevo 2100 system equipped with an ultra-high frequency MS400 cardiovascular transducer (18–38 MHz). Left ventricular (LV) mass and systolic function was assessed at near-physiological heart rates (∼500 bpm) using the short-axis view. Pulsed-wave tissue Doppler imaging of mitral valve flow obtained in apical four-chamber view at lower heart rates (∼350 bpm) was used to evaluate LV diastolic function.

### 2.7. ECG and atrial transoesophageal pacing

Surface ECG was obtained in anaesthetized mice (2% isoflurane) by placing platinum subdermal needle electrodes (F-E7, *Grass Technologies*) in each limb in lead II arrangement. To assess AF susceptibility, the left atrium was paced using an octapolar catheter (CIBer mouse EP catheter, NuMED Inc.) placed in the oesophagus, as described previously.^18^ A decremental burst pacing protocol that consisted of a 40 stimulus drive train at a cycle length of 60 ms with 2 ms decrements until 10 ms was applied. After each burst, the surface ECG was monitored for evidence of atrial arrhythmias and the protocol was continued only after return to sinus rhythm (see also Supplementary material online).

### 2.8. Optical imaging of atrial tissues

Following isolation, atria were incubated for 30 min at 37°C in Tyrode solution containing di-4-ANEPPS (20 µmol/L mixed with DMSO) and blebbistatin (5 µmol/L), after which atria were continuously perfused with Krebs-Henseleit buffer of the following composition (in mmol/L): 118 NaCl, 4.2 KCl, 1.2 KH_2_PO_4_, 1.2 MgSO_4_, 11 NaHCO_3_, 11 glucose, 2 Na-pyruvate, and 1.5 CaCl_2_, supplemented with 1 µmol/L blebbistatin. The solution was continuously bubbled with a 95% O_2_–5% CO_2_ mixture and the temperature was monitored and held at 35–37°C. To excite the dye, illuminating light (545 nm) from an LED source (MacroLED, Cairn Research, UK) was passed through a selection filter and reflected off a dichroic mirror (565 nm, Chroma Technology) onto the preparation. For imaging transmembrane kinetics, emitted light was then passed through a band-pass filter (545/25 nm, Chroma Technology) and captured using a high-speed complementary metal-oxide semiconductor (CMOS) camera (NeuroCMOS-DW128-II, Cairn Research, UK) with a 128 × 128 pixel spatial resolution. The camera was mounted onto an Olympus MVX10 Macroview fluorescent microscope equipped with a 1× or 2× MV PLAPO objective (Olympus). Image sequences of up to 4000 frames were captured at 1000 frames/sec using Gview, a custom-written optical mapping software (courtesy of Professor Gil Bub, University of Oxford). Details of the data analysis are given in the Supplementary material online.

### 2.9. Studies in isolated atrial myocytes

RA and LA myocytes were isolated as described in the Supplementary material online and loaded with Fura-2AM (5 µmol/L, ThermoFisher Scientific). Isolated myocytes, in aliquots of 100 µL, were incubated with ANG-II (1 µmol/L) for a further 20 min before use. Diastolic Ca^2+^ leak from the ryanodine receptor (RyR2) was measured according to a protocol developed by Shannon et al.^19^ and modified for atrial myocytes.^20^ Briefly, myocytes were field-stimulated at 2 Hz at 36 ± 1 ˚C and 1.8 mmol/L extracellular Ca^2+^ until Ca^2+^ transients reached a steady state and were recorded for a further 30 s. Pacing was then stopped, and the bath solution was rapidly changed to a Na^+^-free, Ca^2+^-free Tyrode solution (with Na^+^ replaced by Li^+^) containing tetracaine (1 mmol/L) for 60 s to block RyR2 channel opening. After this period, the solution was changed again, this time to a Na^+^-free, Ca^2+^-free solution without tetracaine for 90 s. Finally, a pulse of caffeine (10 mmol/L) was applied to the myocyte to release Ca^2+^ from the sarcoplasmic reticulum (SR). The RyR2 Ca^2+^ leak was quantified as the tetracaine-dependent change in diastolic Ca^2+^ during the periods of Na^+^-free, Ca^2+^-free Tyrode perfusion. The amplitude of the caffeine-induced Ca^2+^ transient was taken as the SR Ca^2+^ load, which was used to assess the leak–load relationship by plotting the RyR2 Ca^2+^ leak against the SR Ca^2+^ content. The amplitude of the field-stimulated Ca^2+^ transient was calculated by subtracting the baseline (diastolic) Ca^2+^ from the peak (systolic) Ca^2+^ and the time constant (tau, τ) of Ca^2+^ decay by fitting a second order exponential curve to the transient (Axon Clampfit version 8.2.0.233). Measurements from at least 15 s of Ca^2+^ transient recordings were averaged for each cell.

### 2.10. Statistical analysis

All functional studies were performed with investigators blinded of the mice genotype or intervention. Group sizes were determined using GPower 3.1 by power calculations of sample size, applying a power of 80% and *α* = 0.05 (or a 5% significance level). Normality of data sets was assessed using the D’Agostino–Pearson normality test. Normally distributed data are expressed as mean with standard deviation (SD) and analysed using the two-tailed unpaired Student’s *t*-test, one-way ANOVA, or two-way ANOVA with Tukey or Bonferroni correction for multiple comparisons. Non-normally distributed data are expressed as median with inter-quartile range (IQR) and analysed by Mann–Whitney *U* test or Kruskal–Wallis test with Dunn’s correction. Ca^2+^ data were analysed by two-tailed unpaired Student’s *t*-test or Mann–Whitney *U* test using the hierarchical statistical clustering model (lmerTest R package).^21^ The null hypothesis was rejected at a value of *P *<* *0.05.

## 3. Results

### 3.1. Myocardial *NOX2* overexpression increases atrial superoxide and AF susceptibility

**Figure 1:**
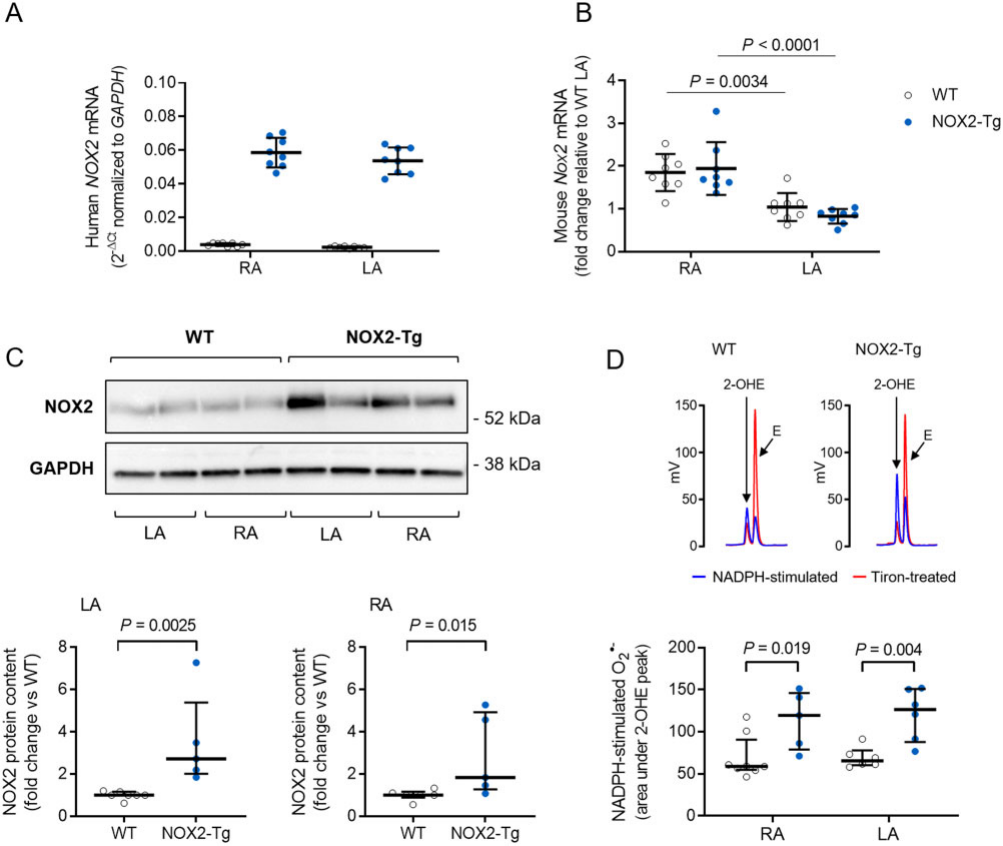
Atrial NOX2 expression and superoxide production in WT and NOX2-Tg hearts. (*A*) Quantitative RT-PCR detection of the human *NOX2* mRNA transcript confirms the presence of the transgene in the LA and RA of NOX2-Tg, but not WT, hearts (*n* = 8/group). (*B*) Expression of mouse *Nox2* mRNA is significantly higher in the RA (*P *=* *0.0034 vs*.* LA-WT and *P *<* *0.0001 vs. LA-NOX2-Tg; one-way ANOVA), with no differences between genotypes. (*C*) Western blot shows detection of a 58 kDa NOX2 protein in atrial tissue homogenates from WT and NOX2-Tg mice. NOX2 protein content is significantly higher in NOX2-Tg hearts in both RA (*P *=* *0.015 vs. WT, Mann–Whitney *U* test, *n* = 6 for WT and *n* = 5 for NOX2-Tg) and LA (*P *=* *0.0025 vs. WT; Mann–Whitney *U* test; *n* = 7 for WT and *n* = 5 for NOX2-Tg) tissue homogenates. Two atria were pooled for each biological replicate. (*D*) Representative HPLC chromatograms showing the formation of 2-hydroxyethidium (2-OHE), the superoxide-derived oxidation product of DHE, and ethidium (*E*), the non-specific two-electron oxidation product, in LA homogenates. The tiron-inhibitable fraction of 2-OHE (i.e. the difference in the area under the 2-OHE peak between NADPH and tiron) was taken as a measure of the NADPH-stimulated superoxide production and found to be significantly higher in NOX2-Tg RA (*P *=* *0.019 vs*.* WT, Mann–Whitney *U* test, *n* = 8 for WT and *n* = 5 for NOX2-Tg) and LA (*P *=* *0.004 vs*.* WT, Mann–Whitney *U* test, *n* = 6/genotype). Graphs show individual data points with means and SDs or medians and IQRs, as appropriate.

Expression of the human *NOX2* cDNA, assessed by quantitative RT–PCR, could be detected in both the LA and RA of NOX2-Tg (*Figure 1A*). As expected, human *NOX2* mRNA was not detected in WT hearts (*Figure 1A*). The expression of murine *Nox2* mRNA was similar between genotypes and was significantly higher in the RA compared with the LA (*Figure 1B*). Atrial NOX2 protein was significantly higher in NOX2-Tg compared with WT mice; NOX2-Tg displayed approximately 2-fold and 2.5-fold higher NOX2 in RA and LA tissue homogenates, respectively, compared with WT (*Figure 1C*). Importantly, functional enzymes were encoded by the human *NOX2* transgene, as indicated by the significantly higher level of NADPH-stimulated superoxide production in both atria of NOX2-Tg (*Figure 1D*).

**Figure 2:**
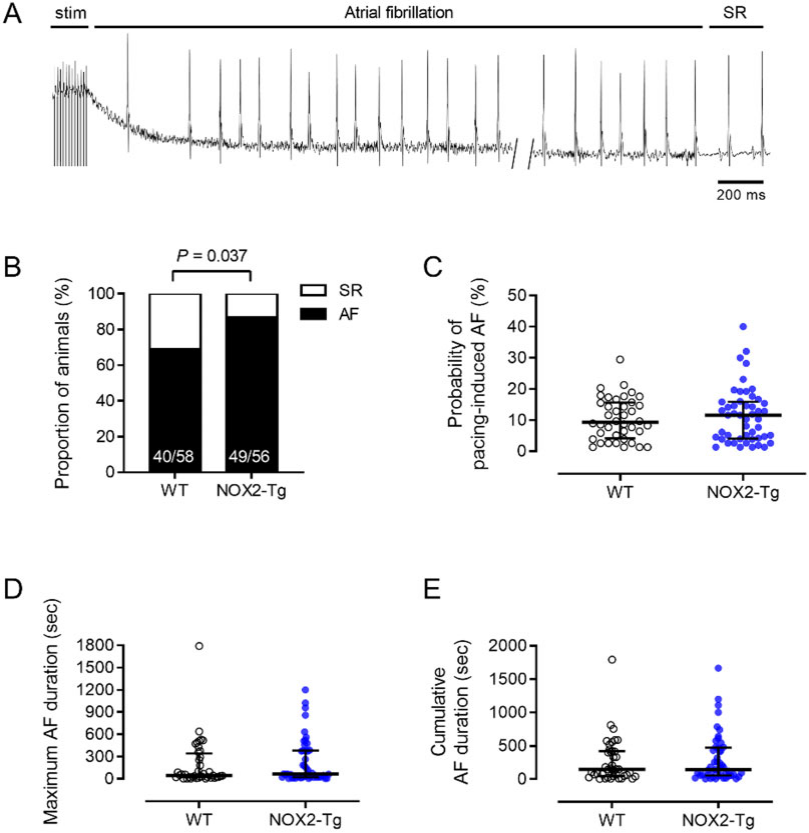
NOX2-Tg mice are more prone to pacing-induced AF. (*A*) Surface ECG recordings illustrate episodes of atrial tachyarrhythmias resembling AF, characterized by absent P-waves and increased R-R interval variance, with recovery of sinus rhythm (SR). (*B*) Percentages of NOX2-Tg and WT mice with inducible AF episodes lasting at least 2 s. Note higher incidence of pacing-induced AF (≥2 s) in NOX2-Tg mice (*P *=* *0.037; *n* = 58 WT and *n* = 56 NOX2-Tg mice). (*C*) The probability of AF induction was not different between genotypes (*P *=* *0.83; *n* = 40 WT and *n* = 49 NOX2-Tg mice). Maximum AF duration (*D*) and cumulative AF duration (*E*) tended to be longer in NOX2-Tg mice but was not statistically significant (*P > *0.05; *n* = 40 WT and *n* = 49 NOX2-Tg mice). Categorical data are shown as percentages and *P-*values were calculated using the Fisher’s exact test. Continuous data are shown as individual data points with medians and IQRs and *P-*values were calculated using the Mann–Whitney *U* test.

LV systolic and diastolic function and mass assessed by echocardiography did not differ significantly between genotypes (*Table 1*). Surface ECG parameters were similar between groups, except for a longer RR interval duration in NOX2-Tg (*Table 1*). Transoesophageal burst pacing uncovered a modest increase in AF susceptibility in NOX2-Tg compared with WT littermates. As documented in *Figure 2* (Supplementary material online, *Table S1*), atrial burst pacing provoked AF (with episodes of rapid atrial activity lasting >2 s) in a greater proportion of NOX2-Tg than WT (88% vs. 69%, respectively, *P* = 0.037; *Figure 2B*), and similar trends were observed when longer bouts of AF (e.g. >5 s and >10) were evaluated (Supplementary material online, *Table S1*). Among those animals with inducible AF, we quantified the probability of AF induction (i.e. the number of AF episodes divided by the total number of burst stimulations delivered to the atria) and the duration of AF episodes (*Figure 2C–E*) and found no significant difference between genotypes (Supplementary material online, *Table S1*).

**Table 1:** Echocardiographic measurements and ECG parameters

	WT	NOX2-Tg	*P*-value
Echocardiography	*N = 20*	*N = 15*	
Body weight (g)	23.2 ± 3.83	23.8 ± 5.48	0.71
LV mass (mg)	85.4 (73.4–109)	100 (89.2–110)	0.15
Indexed LV mass (mg/g)	3.89 (3.30–4.73)	4.41 (3.47–4.76)	0.39
LV EF (%)	71.5 ± 9.68	71.3 ± 8.98	0.95
LV FS (%)	40.7 ± 8.33	40.3 ± 7.42	0.89
Mitral valve *E*/*A* ratio	1.80 (1.5–2.0)	1.77 (1.6–2.0)	0.95
Combined *E*/*E*’ ratio	30.6 ± 6.74	28.8 ± 4.15	0.49
ECG parameters	*n = 63*	*n = 61*	
RR interval (ms)	135 ± 18	142 ± 20	0.037*
P-wave duration (ms)	22.4 (21.2–24.1)	23 (21.6–24.3)	0.32
PQ interval (ms)	40.7 ± 3.27	41.0 ± 3.84	0.67
QRS duration (ms)	8.1 (7.71–8.93)	8.2 (7.18–9.4)	0.58
QTc interval (m)	47.5 ± 6.82	49 ± 7.63	0.23

Data are shown as mean ± SD or median ± inter-quartile range, as appropriate. Normally distributed data were analysed by the two-tailed unpaired Student’s *t*-test whereas non-normally distributed data were analysed by the Mann–Whitney *U* test.

*A*, late mitral valve inflow velocity; *E*, early mitral valve inflow velocity; *E*’, early tissue Doppler mitral valve annulus velocity; EF, ejection fraction; FS, fractional shortening; LV, left ventricle; *N*, number of mice.

Taken together, these findings indicate that myocardial NOX2 overexpression and related increase in NADPH-stimulated superoxide production are sufficient to cause a modest increase in AF susceptibility in the absence of significant changes in AF duration, surface ECG parameters, and LV mass or function.

### 3.2. NOX2-Tg mice display no evidence of atrial electrical or structural remodelling

**Figure 3:**
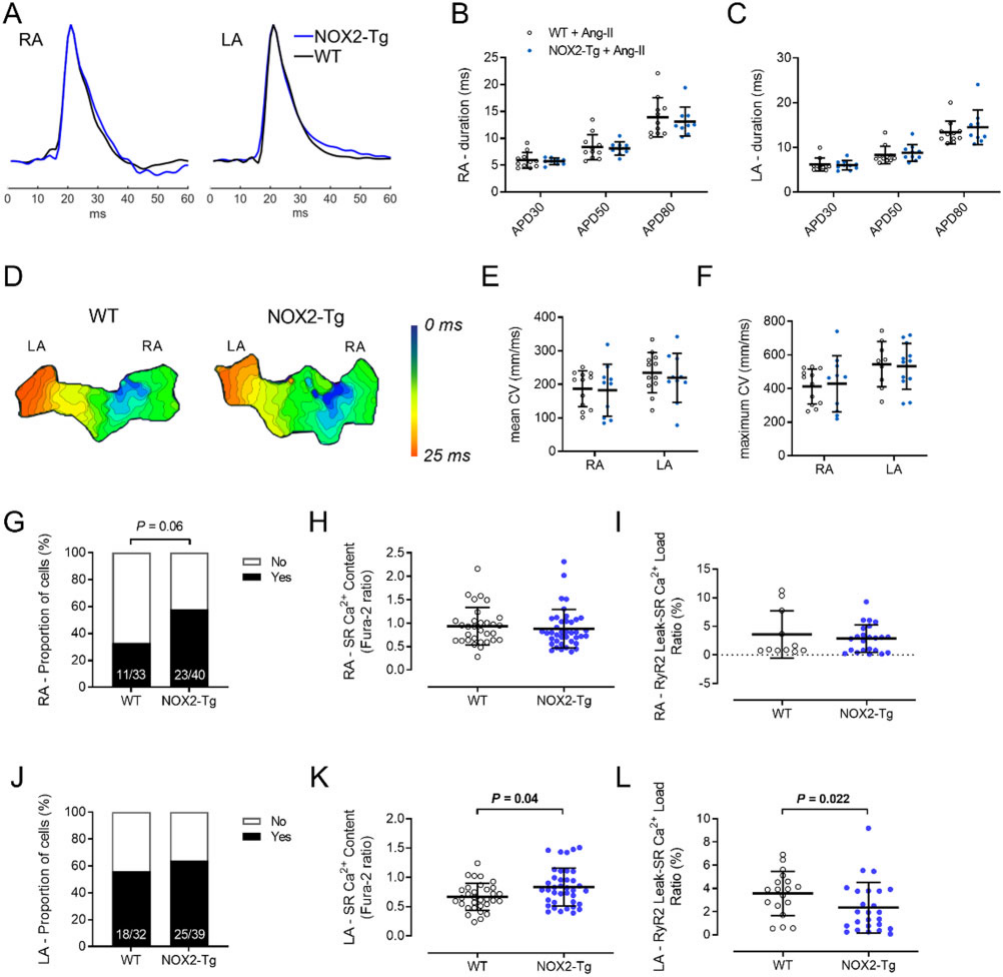
Atrial action potentials, conduction velocity, and diastolic Ca^2+^ leak in WT and NOX2-Tg atria following exposure to ANG-II. (*A*) Representative RA and LA optical action potentials recorded from WT or NOX2-Tg atria incubated with ANG-II. Summarized data for APD30, APD50, and APD80 in the RA (*B*) and LA (*C*) show no significant difference between WT and NOX2-Tg mice. Statistical significance between groups was determined for each APD using unpaired Student’s *t*-test. *n = *11 and 9–10 for WT and NOX2-Tg atria, respectively. (*D*) Representative activation maps of a single spontaneous wave depolarization show a similar pattern of impulse conduction in WT and NOX2-Tg atria. Each isochrone line represents 1 ms. Summarized CV data show no significant difference in mean (*E*; *n* = 12 for WT and *n* = 10 for NOX2-Tg) or maximum (*F*; for RA, *n* = 12 for WT and *n* = 10 for NOX2-Tg; for LA, *n* = 9 for WT and *n* = 12 for NOX2-Tg) CV in the RA or LA between genotypes. Statistical significance between groups was determined for each parameter using unpaired Student’s *t*-test. (*G*) Diastolic Ca^2+^ leak was present in a greater proportion of NOX2-Tg RA myocytes (58%, 23/40 cells) compared with WT controls (33%, 11/33 cells) (*P *=* *0.06, Fisher’s exact). The SR Ca^2+^ content (*H*) and the RyR2 leak—SR load ratio (*I*) was similar between NOX2-Tg (*n* = 23 cells from 9 mice) and WT RA myocytes (*n* = 11 cells from 9 mice). (*J*) The proportion of cells with diastolic Ca^2+^ leak was not different between NOX2-Tg and WT LA myocytes (*P = *0.63, Fisher’s exact). (*K*) SR Ca^2+^ content was significantly greater in NOX2-Tg LA myocytes (*n* = 38 cells from 11 mice; 0.785, 25th–75th percentile: 0.56–1.02) compared with WT (*n* = 32 cells from 8 mice; 0.64, 25th–75th percentile: 0.52–0.81; *P = *0.04), which meant that the RyR2 leak—SR load ratio (*L*) was reduced in NOX2-Tg LA myocytes (F365/380: 1.93, 25th–75th percentile: 0.64–3.78 for NOX2-Tg LA vs. 3.94, 25th–75th percentile: 2.3–4.7 for WT LA, *P *=* *0.022, Mann–Whitney *U* test). Graphs show individual data points with means and SDs or medians and IQRs, as appropriate.

AF occurs when electrical and/or structural abnormalities of the atria promote abnormal impulse formation and propagation.^22^ The hallmark feature of electrical remodelling is a shortening of the atrial refractory period,^23^ while key pathophysiological features of structural remodelling include inflammation and interstitial fibrosis,^24^ changes which impede impulse propagation through the atria and provoke inhomogeneity of electrical conduction and decreased conduction velocity. To gain insight into the possible mechanisms underlying AF in NOX2-Tg mice, we investigated atrial action potential duration (APD) and wavefront conduction velocity using optical imaging of di-4-ANEPPs-stained atrial tissue preparations. Because NOX2 is not constitutively active, *ex vivo* measurements were also taken in the presence of angiotensin-II (ANG-II, 1 µmol/L), a well-characterized activator of NOX2 oxidases.^17^ Optical action potentials were recorded from the RA and LA of NOX2-Tg and WT hearts. Mean APD, measured during spontaneous sinus rhythm, was not statistically different between genotypes either at baseline (Supplementary material online, *Figure S1*) or in the presence of ANG-II (*Figure 3B and C*). Similarly, there were no genotype differences in mean APD recorded during pacing (Supplementary material online, *Table S2*) and premature depolarizations (either early or late after-depolarizations) were never observed.

Furthermore, atrial activation maps showed no differences in conduction patterns between genotypes (*Figure 3D* and Supplementary material online, *Figure S1D*) and there was no effect of NOX2 overexpression on atrial conduction velocity (*Figure 3E and F* and Supplementary material online, *Figure S1E* and *F*). To interrogate for the presence of structural remodelling, we assessed collagen content and the extent of fibroblast differentiation into a myofibroblast phenotype.^25^ α-Smooth muscle actin (α-SMA) protein content was significantly higher in the RA of NOX2-Tg mice (*P* = 0.005 vs*.* WT-RA), whereas no genotype differences were detected in the LA (Supplementary material online, *Figure S2B*). Other markers of myofibroblast differentiation, such as vimentin, did not differ between genotypes in either atrium (Supplementary material online, *Figure S2C*). Importantly, NOX2-Tg atria displayed no excess fibrosis, as *Col1A1* mRNA and total collagen content were similar between genotypes (Supplementary material online, *Figure S2D* and *E*). Furthermore, markers of inflammation were not affected by NOX2 overexpression, as indicated by the similarity in expression of *Il-1β, Il-6,* and *Mcp-1* mRNA in WT and NOX2-Tg atria (Supplementary material online, *Figure S2F–H*).

Abnormalities in intracellular Ca^2+^ handling also feature prominently in AF and increased diastolic Ca^2+^ leak from the SR via the RyR2 has been proposed as a mechanism contributing to AF initiation.^26^^,^^27^ To investigate SR Ca^2+^ leak in atrial myocytes of WT and NOX2-Tg mice, we used the method developed by Shannon et al.,^19^ where SR Ca^2+^ leak is quantified directly as the tetracaine-inhibitable increase in cytosolic Ca^2+^ when *trans*-sarcolemmal Ca^2+^ fluxes are blocked by a Na^+^-free, Ca^2+^-free extracellular solution. LA and RA cardiomyocytes were exposed to ANG-II (1 µmol/L for 20 min) and Ca^2+^ transients were evoked by field stimulation at 2 Hz. We observed no significant differences in diastolic Ca^2+^, Ca^2+^ transient amplitude, and time constant of Ca^2+^ decay (tau, τ) between WT and NOX2-Tg (Supplementary material online, *Figure S3*). Further, analysis of the caffeine-induced Ca^2+^ transients revealed no significant genotype differences in the SR Ca^2+^ load of RA myocytes (*Figure 3H*) but a modest increase SR Ca^2+^ content in LA myocytes from NOX2-Tg vs. WT (*P* = 0.004; *Figure 3K*).

A Ca^2+^ leak was quantifiable in a greater proportion of RA myocytes from NOX2-Tg compared with WT (11/33 cells (33%) for WT vs*.* 23/40 cells (58%) for NOX2-Tg; *P* = 0.06; *Figure 3G*), but the ratio of RyR2 Ca^2+^ leak to SR Ca^2+^ load was not different between genotypes (*Figure 3I*).

In the LA, the proportion of myocytes with SR Ca^2+^ leak was similar between genotypes (18/32 (56%) in WT vs*.* 25/39 (64%) in NOX2-Tg; *Figure 3J*)—although relative to RA the general proportion of cells with SR Ca^2+^ leak was higher—while the ratio of RyR2 leak to SR Ca^2+^ load was significantly smaller in NOX2-Tg compared with WT (*Figure 3L*).

As RyR2 activity is influenced by the prevailing myocardial redox state, either by direct oxidation of cysteine thiol residues^28^ or indirectly through phosphorylation by the protein kinases PKA^29^ and CaMKII,^30^ we next investigated whether changes in RyR2 phosphorylation may account for the differences in RyR2 Ca^2+^ leak observed between NOX2-Tg and WT LA myocytes. As shown in Supplementary material online, *Figure S4A and B*, phosphorylation of RyR2-Ser2808 and RyR2-Ser2814 was similar between genotypes in both RA and LA homogenates. Furthermore, no difference in CaMKII phosphorylation at threonine 287 or oxidation (Supplementary material online, *Figure S5A* and *B*) was observed between WT and NOX2-Tg atrial tissues.

Overall, these findings indicate a lack of significant atrial substrate promoting AF induction in NOX2-Tg mice, in line with our finding that NOX2-Tg has only a modest increase in AF susceptibility in the absence of differences in probability of AF induction and AF duration.

### 3.3. ATV attenuates atrial superoxide production in NOX2-Tg mice without modifying the AF phenotype

**Figure 4:**
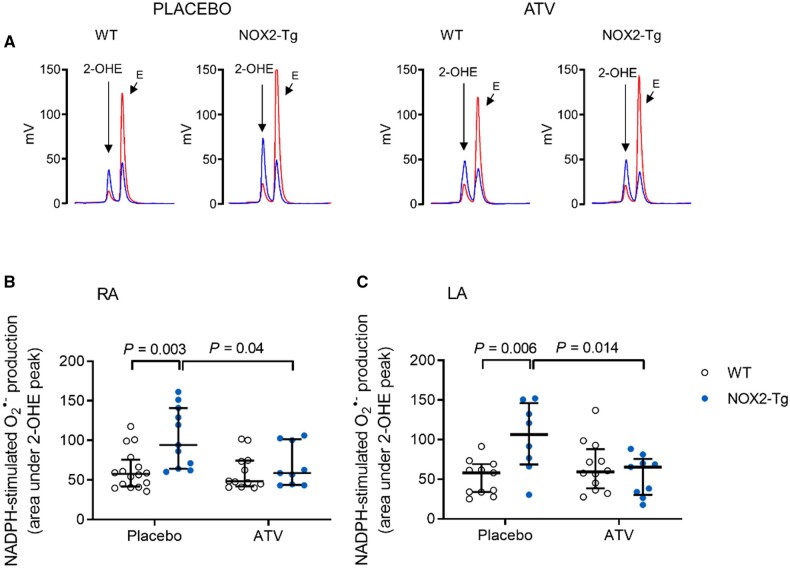
Atorvastatin prevents the increase in NADPH-stimulated atrial superoxide production secondary to NOX2 overexpression. (*A*) Representative HPLC chromatograms of superoxide production (2-OHE peak) in LA homogenates from WT and NOX2-Tg mice given ATV or placebo diet are shown. *Blue*, NADPH-stimulated superoxide production; *Red*, Tiron-inhibited trace. (*B*) NADPH-stimulated superoxide production is higher in RA homogenates of placebo-treated NOX2-Tg mice (*P *=* *0.003 vs*.* WT-placebo). This difference is abolished by ATV treatment (*P *=* *0.9). For placebo, *n* = 16 for WT and *n* = 11 for NOX2-Tg; and for ATV, *n* = 12 for WT and *n* = 9 for NOX2-Tg. (*C*) The increase in NADPH-stimulated superoxide production in LA homogenates of placebo-treated NOX2-Tg mice (*n* = 8) compared with WT controls (*n* = 11, *P *=* *0.006) is prevented by ATV treatment, with no differences between ATV-treated groups (*P *=* *0.84). For placebo, *n* = 11 for WT and *n* = 8 for NOX2-Tg; and for ATV, *n* = 12 for WT and *n* = 9 for NOX2-Tg. Two-way ANOVA with Tukey *post hoc* analysis was used to determine statistical significance of placebo and superoxide production with ATV between groups. Graphs show individual data points with medians and IQRs. For these experiments, two atria were pooled for each biological replicate.

We then investigated whether statin therapy reduces atrial NOX2-derived superoxide production and AF inducibility in mice. To that end, we allocated WT and NOX2-Tg mice to ATV (30 mg/kg/day, *p.o.*) or placebo for 2 weeks. ATV did not affect food consumption, body weight, or myocardial and plasma cholesterol content (Supplementary material online, *Figure S6A–D*). NADPH-stimulated superoxide production in RA and LA homogenates from ATV-fed NOX2-Tg mice was significantly lower than that measured in placebo-fed NOX2-Tg mice (*Figure 4*); in contrast, ATV had no effect on atrial superoxide production in WT mice. As a result, genotype differences in atrial superoxide production were abolished in ATV-treated mice. We also measured the expression of human (Supplementary material online, *Figure S7A*) and murine (Supplementary material online, *Figure S7B*) *NOX2* mRNA and found it to be unchanged by ATV treatment, suggesting that the effect of ATV on atrial NADPH-stimulated superoxide production occurs through its inhibitory action on the NOX2 enzymatic complex.

**Figure 5:**
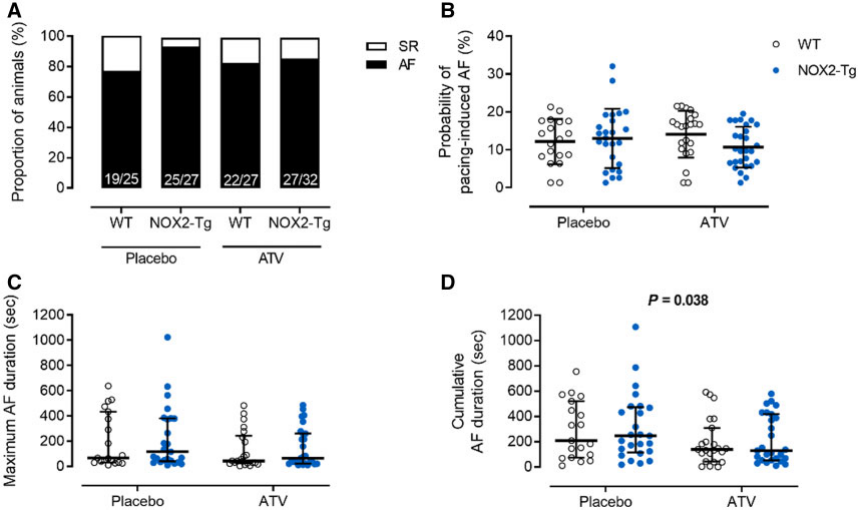
Pacing-induced AF susceptibility in placebo and atorvastatin-treated WT and NOX2-Tg mice. (*A*) Percentages of NOX2-Tg and WT mice with inducible AF episodes lasting at least 2 s (*n* numbers as indicated). (*B*) The probability of AF (≥2 s) induction per mouse was not different between genotypes (*P *=* *0.83) and there was no effect of ATV (*P *=* *0.89). (*C*) Maximum AF durations tended to be longer in placebo-fed NOX2-Tg mice (117 s, 25th–75th percentile: 40–381 vs*.* 68 s, 25th–75th percentile: 32–433 s for placebo-fed WT) and shorter with ATV treatment (*P *=* *0.07; WT, 43 s, 25th–75th percentile: 25–242 s vs*.* NOX2-Tg, 65 s, 25th–75th percentile: 22–261 s). (*D*) Cumulative AF duration was significantly lower in mice who received ATV (*P *=* *0.038 vs*.* placebo), independent of genotype. AF probability and durations were quantified from mice that developed AF lasting at least 2 s (for placebo, *n* = 19 WT and *n* = 25 NOX2-Tg mice; for ATV, *n* = 22 WT and *n* = 27 NOX2-Tg mice). Categorical data are shown as percentages and *P-*values were calculated using the Fisher’s exact test. Continuous data are shown as individual data points with means and SDs or medians and IQRs and *P-*values were calculated by two-way ANOVA with Tukey *post hoc* analysis. To achieve normality, non-parametric data were log-transformed before statistical analysis.

AF susceptibility was not significantly affected by ATV treatment (*Figure 5A* and Supplementary material online, *Table S3*); among those animals with inducible AF (76% of WT and 93% of NOX2-Tg mice receiving placebo; 81% of WT and 84% NOX2-Tg receiving ATV), the probability of AF induction (*Figure 5B*) and maximum AF duration (*Figure 5C*) was similar in all four groups. Similarly, ATV treatment had no effect on LV structure and function or on ECG parameters (Supplementary material online, *Table S4*). ATV was found, however, to significantly lower cumulative AF duration (*P* = 0.038, *Figure 5D*), but this effect occurred to a similar degree in both genotypes indicating this was independent of ATV’s ability to lower NOX2-derived superoxide production.

## 4. Discussion

In the present study, we demonstrate that atrial NOX2 overexpression leads to a modest increase in AF inducibility in mice without impacting on AF duration or probability. NOX2-derived superoxide seems unlikely to be the primary driver of AF development, as treatment with ATV, which normalized NOX2-derived superoxide production in NOX2-Tg mice, did not significantly reduce AF induction. Our data further show that the NOX2-dependent increase in AF susceptibility occurs in the absence of electrical and structural remodelling but is instead associated with modest RyR2 dysregulation. Together, these data show that while atrial NOX2 overexpression may contribute to atrial triggered activity underlying arrhythmogenesis, NOX2-derived superoxide production is not crucial to the maintenance of AF and is more likely a biomarker of AF-induced remodelling than causal in its development.

### 4.1. Myocardial NOX2 overexpression increases AF inducibility

AF has been consistently associated with atrial oxidative stress.^31^ Among the different sources of ROS in the myocardium, NOX2-containing NADPH oxidases contribute significantly to the production of superoxide in human atrial myocytes.^7^ Previous work has demonstrated that a roughly twofold increase in NOX2-mediated superoxide accompanies AF in patients^7^^,^^10^ and animal models of pacing-induced AF^10^ and further showed a strong independent association between atrial NOX2 activity and post-operative AF in patients undergoing cardiac surgery.^14^^,^^15^ Nevertheless, direct experimental evidence linking NOX2 activity to the initiation and/or maintenance of AF has always been lacking.

By using a NOX2 transgenic mouse model, which produces superoxide to an extent marginally comparable to that observed in patients who develop post-operative AF,^10^ we were able to show that up-regulation of atrial NOX2 is sufficient to increase AF induction in response to burst pacing. Notwithstanding, we did not observe any difference in AF duration between NOX2-Tg and WT, which suggests that NOX2 may be involved in the initiation rather than maintenance of AF. This conclusion is in line with previous work by Reilly et al.^10^ which showed that atrial NOX2 activity is transiently up-regulated early after AF induction in goats but is not a major contributor to atrial superoxide levels in human or experimental long-standing AF. In particular, RA NOX2-mediated superoxide production was shown to be increased in paroxysmal AF and predictive of post-operative AF in patients in sinus rhythm at the time of cardiac surgery.^10^^,^^13–15^

### 4.2. Putative cellular and molecular mechanisms of AF in NOX2-Tg

Our present understanding of AF causation is that ectopic atrial activity, mediated by abnormal Ca^2+^ handling, acts as a trigger, while a permissive tissue substrate supports the maintenance of a sustained arrhythmia.^22^ While alterations in myocardial NOX2 activity have been closely linked to AF,^31^ the precise mechanisms mediating this association are poorly defined. APD shortening, a prominent feature of atrial electrical remodelling sustaining AF, was not observed in NOX2-Tg atria. Moreover, the absence of fibrosis, despite indications of myofibroblast activation (i.e. increased α-SMA) in the RA, or changes in mean CV also suggests that atrial NOX2 overexpression is unlikely to induce significant structural remodelling promoting AF.

In the ventricular myocardium, there is evidence that NOX2-derived ROS may increase cardiac RyR2 open probability leading to aberrant Ca^2+^ release and arrhythmias.^32^ NOX2 is expressed in sarcolemmal/t-tubule membranes of ventricular myocytes, in close proximity to RyR2 channels,^33^ where it has been reported to promote diastolic SR Ca^2+^ leak and cellular arrhythmias by activating CaMKII.^32^ Although we cannot completely exclude a link between NOX2 and RyR2 function, any effects of NOX2 are unlikely to be mediated by PKA- or CaMKII-dependent phosphorylation of RyR2, as we see no changes in RyR2 phosphorylation at the PKA/CaMKII sites, Ser-2808 and Ser-2814 with NOX2 overexpression. NOX2-derived superoxide may also lead to direct redox modification of RyR2, resulting in increased Ca^2+^ leak.^28^ Although a Ca^2+^ leak was detectable in a greater proportion of RA myocytes from NOX2-Tg compared with WT, the SR Ca^2+^ leak-to-load ratio was similar between genotypes in RA myocytes and slightly reduced in NOX2-Tg LA myocytes, disputing the presence of direct, NOX2-dependent effect on RyR2 open probability in NOX2-Tg.

### 4.3. Effect of statin therapy on atrial NOX2-dependent superoxide production and AF induction

Perioperative statin therapy has been shown to reduce superoxide production by NOX2-containing NADPH oxidases in human atrial tissues by inhibiting Rac1 activity.^10^^,^^15^ Our observation that oral ATV treatment significantly attenuated atrial superoxide generation in NOX2-Tg but not WT atria is consistent with these reports and suggests that statins may have potential utility in preventing AF in conditions, such as haemodynamic stress and inflammation, where NOX2 activity is elevated ^34^. While a number of small clinical trials reported a reduction in the new onset of AF after cardiac surgery in patients allocated to perioperative statin therapy^16^, a much larger randomized controlled trial of perioperative statin therapy showed no discernible benefit of rosuvastatin on the incidence of AF after cardiac surgery,^16^ in keeping with our findings showing that inhibition of NOX2 activity by ATV does not lead to a reduction in pacing-induced AF in NOX2-Tg or WT. Nevertheless, our results uncovered a significant reduction in the duration of AF in both genotypes, suggesting that statins may reduce AF burden, albeit independent of NOX2 inhibition.

### 4.4. Limitations

Although our data show no direct evidence for NOX2 activity in the development of AF, there are several limitations to our study. Because of the methodology used in our Ca^2+^ leak experiments, we were not able to quantify Ca^2+^ leak from a large proportion of myocytes (∼50% of all myocytes studied) meaning we were only powered to detect large differences. NOX2 activation has been reported to increase the diastolic SR Ca^2+^ leak, measured by Ca^2+^ sparks, in ventricular myocytes,^32^ and thus, the use of more sensitive techniques may provide better insights into the potential link between NOX2 and RyR2 open probability in atrial myocytes.

The dose of ATV used in our study (30 mg/kg/day) was substantially higher than that given to patients (40–80 mg daily).^16^ Regardless, even at this high dose and in the presence of a significant reduction in NOX2-derived superoxide, AF susceptibility was not reduced by treatment with ATV.

## 5. Conclusions

Our findings do not support a role for myocardial NOX2-derived superoxide in promoting atrial electrical or structural remodelling or in the causation and maintenance of AF.

## Supplementary material

Supplementary material is available at *Cardiovascular Research* online.

## Data availability

The data underlying this article will be shared on reasonable request to the corresponding author.

## Authors’ contributions

B.C. conceived the study. A.S.M, A.R., J.N.S., and R.C. performed experiments, refined the experimental protocol, and analysed the data. J.N.S. and B.C. supervised the research. G.B. and S.V. set up the equipment and wrote the scripts for the acquisition of optical imaging and transoesophageal pacing data, respectively. K.Z. and J.T. wrote the scripts for analysing optical imaging data. A.C.B and A.M.S generated the mouse model. A.S.M, J.N.S., and B.C. wrote the manuscript. All authors discussed the data and edited the manuscript.

## Supplementary Material

cvab019_Supplementary_Data

## Notes

Translational perspective Atrial NOX2-derived superoxide is an independent predictor of post-operative AF in humans and contributes to atrial oxidative stress early after AF induction. While experimental models have reported an association of NOX2 with AF-induced remodelling, a causal link between NOX2 activity and AF remains to be established. Here, we show that overexpression of the human NOX2 gene in mice resulted in a twofold higher atrial superoxide production and a modest increase in AF susceptibility, independent of atrial electrical or structural remodelling. Short-term treatment with atorvastatin normalized atrial superoxide in NOX2-Tg mice without affecting AF susceptibility. Together, these findings indicate that atrial NOX2-derived superoxide is more likely a biomarker of AF risk than a primary driver of AF development, and that NOX2 inhibition is unlikely to prevent the new onset of AF.
