# Supplementary material for: Inducibility, but not stability, of atrial fibrillation is increased by NOX2 overexpression in mice
Source: Cardiovasc Res. 2021 Jan 23;117(11):2354–64. doi: 10.1093/cvr/cvab019 (PMC8479801; doi:10.1093/cvr/cvab019)
Supplement: cvab019_Supplementary_Data [file cvab019_supplementary_data.pdf]

SUPPLEMENTARY MATERIAL

Inducibility, but not stability, of atrial fibrillation is increased by NOX2 overexpression in mice

Alexandra S. Mighiu[1*], Alice Recalde[1*], Klemen Ziberna[1], Ricardo Carnicer[1], Jakub Tomek[2], Gil Bub[2], Alison C. Brewer[3], Sander Verheule[4], Ajay M. Shah[3], Jillian N. Simon[1†], Barbara Casadei[1†]

[1]Division of Cardiovascular Medicine, University of Oxford, UK
[2]Department of Physiology, Anatomy and Genetics, University of Oxford, UK
[3]King's College London British Heart Foundation Centre of Excellence, School of Cardiovascular Medicine & Sciences, London, UK
[4]Department of Physiology, Maastricht University, Maastricht, Netherlands

** these authors contributed equally*
† these authors contributed equally to the supervision of this work

Short title: NOX2 activity is not causal for atrial fibrillation

Address for correspondence:

Professor Barbara Casadei
Division of Cardiovascular Medicine
Radcliffe Department of Medicine
University of Oxford
L6, West Wing
John Radcliffe Hospital
Oxford, OX3 9DU
UK
Email: barbara.casadei@cardiov.ox.ac.uk
Phone: +44(0)1865 234664

Dr Jillian N. Simon
Division of Cardiovascular Medicine
Radcliffe Department of Medicine
University of Oxford
L6, West Wing
John Radcliffe Hospital
Oxford, OX3 9DU
UK
Email: jillian.simon@cardiov.ox.ac.uk
Phone: +44 01865 234902

SUPPLEMENTARY METHODS

RNA isolation and quantitative reverse transcription PCR

Mice were culled by Schedule 1 cervical dislocation and the thorax was rapidly opened to expose the ventricles and surrounding tissues. WT and NOX2-Tg hearts were perfused with ice-cold PBS and the atria and left ventricle were carefully dissected and frozen at -80 °C. Tissues were homogenized using pre-cooled stainless-steel beads and a small bead mill (TissueLyser LT, Qiagen) and total RNA was isolated using the mirVana RNA isolation kit (ThermoFisher Scientific). RNA concentration was determined by measuring the absorbance at 260 nm using the NanoDrop ND-1000 Spectrophotometer (ThermoFisher Scientific), normalised to a standard concentration (25 ng/µL) and stored at -80 °C to prevent degradation until further processing. cDNA was synthesized from 300 ng total RNA with the QuantiTect Reverse Transcription Kit (Qiagen), according to the manufacturer's instructions. For the PCR reaction, 4.5 µL of cDNA (10 ng) was mixed with 5 µL of the TaqMan Fast Advanced Master Mix (ThermoFisher Scientific) and 0.5 µL of the appropriate TaqMan probe in a 96 or 384-well plate. Plates were sealed with Optical Adhesive Covers (ThermoFisher Scientific) and centrifuged briefly to ensure that the entire volume was at the bottom of the wells. Each sample was assayed in duplicates, including negative controls containing RNase free water instead of cDNA. Housekeeping genes were included in each plate to control for variability between samples. All reactions were performed using the ABI Prism system (Applied Biosystems) and quantitative analysis was performed using the comparative Ct method (ΔΔCt method) with reference to a housekeeping gene, as described in User Bulletin No. 2: Relative Quantitation of Gene Expression (Applied Biosystems).

Measurement of superoxide production

For each biological replicate, frozen RA and LA tissues were pooled from two different mice and samples were homogenized in Krebs-HEPES buffer (in mmol/L: 118 NaCl, 10 HEPES, 25

$NaHCO_3$, 5.6 glucose, 4.7 KCl, 1.2 KH_2PO_4, 1.1 $MgSO_4$, 1.4 $CaCl_2$, pH 7.4) supplemented with tablet protease inhibitors (ThermoFisher Scientific). Homogenates were centrifuged at 13,000 rpm for 5 minutes (4°C) and the protein content in the supernatant fraction was measured using the BCA protein assay kit (ThermoFisher Scientific). Samples were then diluted to obtain a final concentration of 100 µg/mL. An aliquot (100 µL) of each sample was then transferred to a separate amber tube and incubated with NAPDH (100 µmol/L) for 15 minutes at 37°C. A separate aliquot of equal volume was incubated with NADPH for 15 minutes at 37°C followed by tiron (100 mmol/L) for an additional 15 minutes at 37°C. Both aliquots were then incubated with dihydroethidium (DHE, 50 µmol/l) for 15 minutes at 37°C. After incubation, samples were further processed for high-performance liquid chromatography analysis by adding methanol and HCl (0.1 mol/L).

A portion of each sample (150 µL) was then injected into an isocratic HPLC system equipped with a Jasco PU-2080 pump, Jasco X-LC 3075 UV absorbance detector and a Jasco FP-2020 Plus fluorescence detector. DHE was monitored with UV absorption at 355 nm; 2-hydroxyethidium (2-OHE) and ethidium production was monitored by the fluorescence detector with excitation at 480 nm and emission at 590/595 nm. The mobile phase was composed of 0.1% trifluoroacetic acid and an acetonitrile gradient (from 30% to 50% over 23 minutes) at a flow rate of 1.0 mL/min. Quantification of superoxide was done by comparing the area under the 2-OHE peak between NADPH-and tiron-treated sample and normalizing the signal to the protein content. All results are expressed as the tiron-inhibitable fraction of 2-OHE as this measurement is taken to be representative of the true level of superoxide production.

ECG and AF induction protocol

Surface ECG was obtained by connecting limb needle electrodes to an Iso-DAM8A amplifier (World Precision Instruments) and CED Power 1401-3A interface (Cambridge Electronic Design Ltd.). Data were acquired using the Spike 2 electrophysiology software. ECG signals were sampled at 2000 Hz and displayed in real time. After placement of the needle electrodes, mice were given a minimum of 5 minutes for the heart rate to stabilize. Standard ECG parameters were measured and averaged from five consecutive beats: R-R interval, P-Q interval, P wave duration, QRS duration, and corrected QT interval. The corrected QT interval (QTc) was calculated as per Bazett's formula (modified for the mouse): $QTc = QT/\sqrt{(RR/100)}$.

AF was induced by atrial burst pacing via a transoesophageal octapolar catheter. First, the catheter was inserted into the oesophagus and placed at the location where the amplitude of the atrial signal was maximal. The diastolic pacing threshold was then determined by pacing the atria at a basic cycle length that was 10- to 20-ms under the sinus cycle length (typically 100-ms) while slowly changing the position of the catheter and the amperage to find the position with the lowest atrial capture threshold (i.e., the lowest voltage required to achieve 1:1 conduction to the ventricles), which also indicates correct catheter placement at the atria. All subsequent stimulations were delivered at twice the threshold amperage with a stimulus duration of 1-ms. The occurrence of AF was identified from the surface ECG by the development of rapid and irregular atrial rhythms, often characterised by the absence of regular P waves, and irregular ventricular activation. AF was then analysed based on arrhythmia duration, initially applying a cut-off of 2-sec and subsequently 5-sec and 10-sec. If burst electrical stimulation provoked arrhythmias that were less than the specified cut-offs or did not evoke an arrhythmic episode at all, the mouse was said to be in sinus rhythm. AF vulnerability was assessed by quantifying the incidence (proportion of mice that developed an episode of

AF) and probability (number of arrhythmic episodes divided by the total number of testing manoeuvres applied) of pacing-induced AF. AF duration was measured from the end of the pacing train until the first sinus P wave and maximum (the longest AF episode in each animal), cumulative (cumulative sum of all discrete AF episodes in each animal), and mean (average of all discrete AF episodes in each animal) AF durations were quantified and compared between groups.

Atrial optical imaging

Data collection and offline analysis

Image sequences were processed using the GView software. The graphical interface allows users to select regions of interest on the image and generates a time series of optical action potentials that is exported as plain text. Optical action potentials were obtained from five different regions (12-by-12 pixels) and APD values were computed from inverted fluorescence data using custom-written MATLAB scripts (version R2015a, Mathworks) that were developed based on the methods and recommendations by Laughner et al.[35] Briefly, baseline drift in the raw optical recordings was corrected by fitting a 5th-order polynomial and subtracting that from the raw signal to establish a constant baseline level. A bi-directional filter (5th order Butterworth filter) with a 100 Hz cut-off was then applied to the fluorescence trace to reduce high frequency noise (>100 Hz) and increase the signal-to-noise ratio. Next, individual action potentials were identified using the *findpeaks* function which locates the local maxima within the trace. To ensure the software did not detect unwanted peaks buried in noise, a minimum peak interval and amplitude was defined. Action potentials were then analysed to determine the amplitude, and activation and repolarization times. The amplitude was given by the difference between the maximum value and the baseline of the fluorescence trace which was an average of 50 frames preceding the action potential upstroke. The activation time was

determined as the time of the first derivative of the fluorescence signal (dF/dt) corresponding to the steepest segment, or the maximum slope, of the action potential upstroke. Repolarization time was measured as a time when a specified level of repolarization (30%, 50% and 80% [APD30, APD50, and APD80, respectively]) was reached during the repolarization phase of the action potential. APD was calculated as the difference between repolarization and activation times and averaged to give an estimate of the global APD in each atrium.

Epicardial conduction velocities were analysed from optical image sequences using the *Ccoffinn* toolkit implemented in MatLab (courtesy of Jakub Tomek, University of Oxford).[36] *Ccoffinn* is a tool that constructs a representation of cardiac waves and tracks their movement, using that information to describe the properties of the observed waves. Briefly, on a frame by frame basis, a peak-finding algorithm identifies the location of pixels that are activated in each frame of the recording (to estimate which parts of the tissue are firing in a synchronized manner) and groups them together as wavefronts. From this, waves are detected frame by frame and the movement of wavefronts is tracked throughout the recording. The tracking information (speed, distance, and direction) is then used to extract the conduction velocity (CV) of each wavefront. The mean CV, taken as the average of all wavefronts in the activation sequence, and maximum CV, taken as the fastest wavefront in the activation sequence, were calculated and averaged for each atrium during sinus rhythm.

Isolation of atrial myocytes

Mice were injected with heparin (1000 U/mL, *ip*) and sacrificed by Schedule 1 cervical dislocation. The thorax was opened by a midsternal incision and the heart was dissected out and rapidly immersed in a Ca^{2+}-free Tyrode solution (in mmol/L: 130 NaCl, 5.6 KCl, 3.5 $MgCl_2$, 5.0 HEPES, 0.4 Na_2PO_4, 20 taurine, 10 glucose, with pH adjusted to 7.4 with NaOH).

The lungs, thymus, and fat were carefully removed to expose the aorta which was subsequently inserted onto a cannula (made from a blunted 23 gauge needle) filled with the Ca^{2+}-free solution. A surgical suture thread (Ethicon 5/0 silk) was looped around the aorta and tightened in order to secure the cannula. A second suture was added to ensure adequate filling and perfusion of the atria and the aorta was then cannulated onto a Langendorff perfusion apparatus.

The heart was perfused with a Ca^{2+}-free Tyrode solution (maintained at 37 °C) for 3 minutes to clear the blood, and then with a primary enzyme solution (containing 1 mg/mL collagenase type II, 0.233 mg/mL protease, 0.166% BSA, and 50 µmol/L Ca^{2+} in Tyrode solution) for a further 8-10 minutes until the heart was digested. At the end of this period, the heart was detached from the cannula and the left and right atria were separated from the ventricles. Each atrium was cut into several pieces and transferred into individual 15 mL cell culture tubes with 500 µL Kraft-Brühe buffer (in mmol/L: 70 glutamic acid potassium salt, 25 KCl, 20 taurine, 20 KH_2PO_4, 3 $MgCl_2$, 0.5 EGTA, 10 glucose, and 10 HEPES). Atrial tissue pieces were gently triturated for 3 minutes in a water bath (37 °C) to disperse the atrial myocytes. The presence of atrial cardiomyocytes was confirmed by microscopic visualization and freshly isolated myocytes were stored at room temperature until experiments were performed later the same day.

SUPPLEMENTARY FIGURES

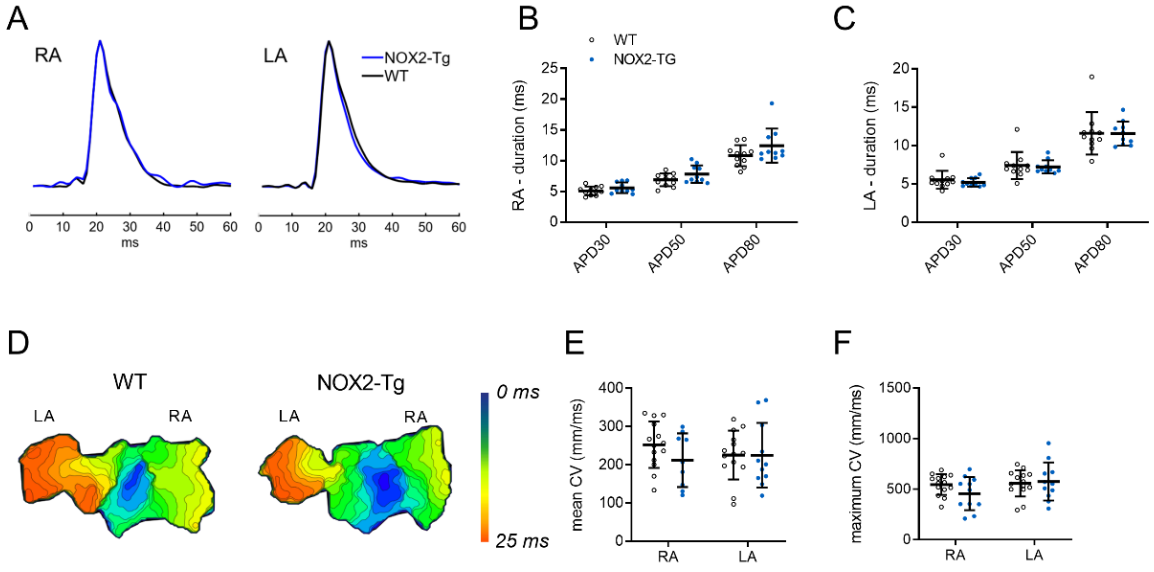

Supplementary Figure 1: Baseline atrial action potentials and conduction velocity in WT and NOX2-Tg atria. (**A**) Representative RA and LA optical action potentials measured at baseline in WT and NOX2-Tg atria. Summarized data for APD30, APD50 and APD80 in the RA (**B**) and LA (**C**) shows no significant difference between WT and NOX2-Tg mice. (n=11 for WT and n=10 for NOX2-Tg). (**D**) Representative activation maps of a single spontaneous wave depolarization shows a similar pattern of impulse conduction in WT and NOX2-Tg atria. Each isochrone line represents 1-ms. Summarized CV data shows no significant difference in mean (**E**) or maximum (**F**) CV in the RA or LA between genotypes (n=14 for WT and n=11 for NOX2-Tg). The unpaired Student's t-test was used for comparisons between genotypes.

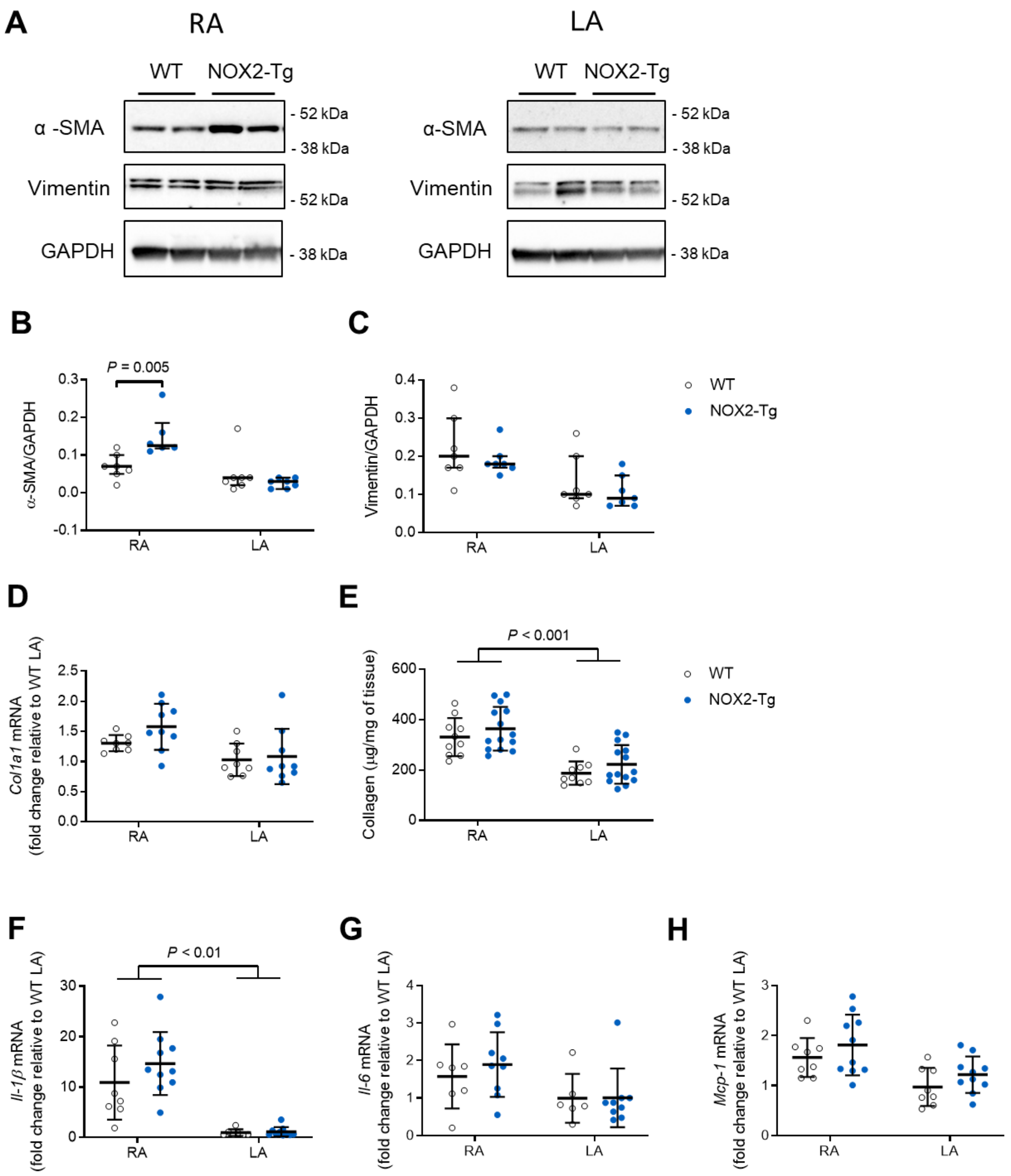

Supplementary Figure 2: Atrial fibrosis and inflammation is unchanged in NOX2-Tg mice. (**A**) Representative Western blots for myofibroblast markers (α-SMA and vimentin) in RA and LA tissue from both genotypes. (**B**) α-SMA protein content is significantly higher in the RA of NOX2-Tg hearts (P=0.005 *vs* WT-RA; n=7 WT and n=6 NOX2-Tg atria), with no genotype differences in the LA (n=7 WT and n=7 NOX2-Tg atria). (**C**) Atrial expression of vimentin was found to be similar between WT and NOX2-Tg atria (n=7 for WT and n=7 for NOX2-Tg). The Mann-Whitney *U* test was used for comparisons between genotypes. Despite indices of myofibroblast activation, atrial collagen 1A1 mRNA expression (n=8-9 per genotype; **D**) and total collagen content (n=9-14 per genotype; **E**) show

no genotype differences, but collagen content is significantly higher in the RA *vs* LA of both WT and NOX2-Tg mice ($P<0.001$, one-way ANOVA with Tukey post-hoc comparison). Atrial mRNA expression of inflammatory markers *Il-1β* (**F**), *Il-6* (**G**) and *Mcp-1* (**H**) is not significantly different between NOX2-Tg and WT mice (n=6-10 per genotype) but expression of *Il-1β* mRNA is significantly higher in the RA *vs* LA of both WT and NOX2-Tg mice ($P<0.05$, Kruskal Wallis test with Dunn's post-hoc comparison). Graphs show individual data points with means and SDs or medians and IQRs, as appropriate. α-SMA – α-smooth muscle actin; IL-1β – interleukin-1β; IL-6 – interleukin-6; MCP-1 – monocyte chemoattractant protein 1.

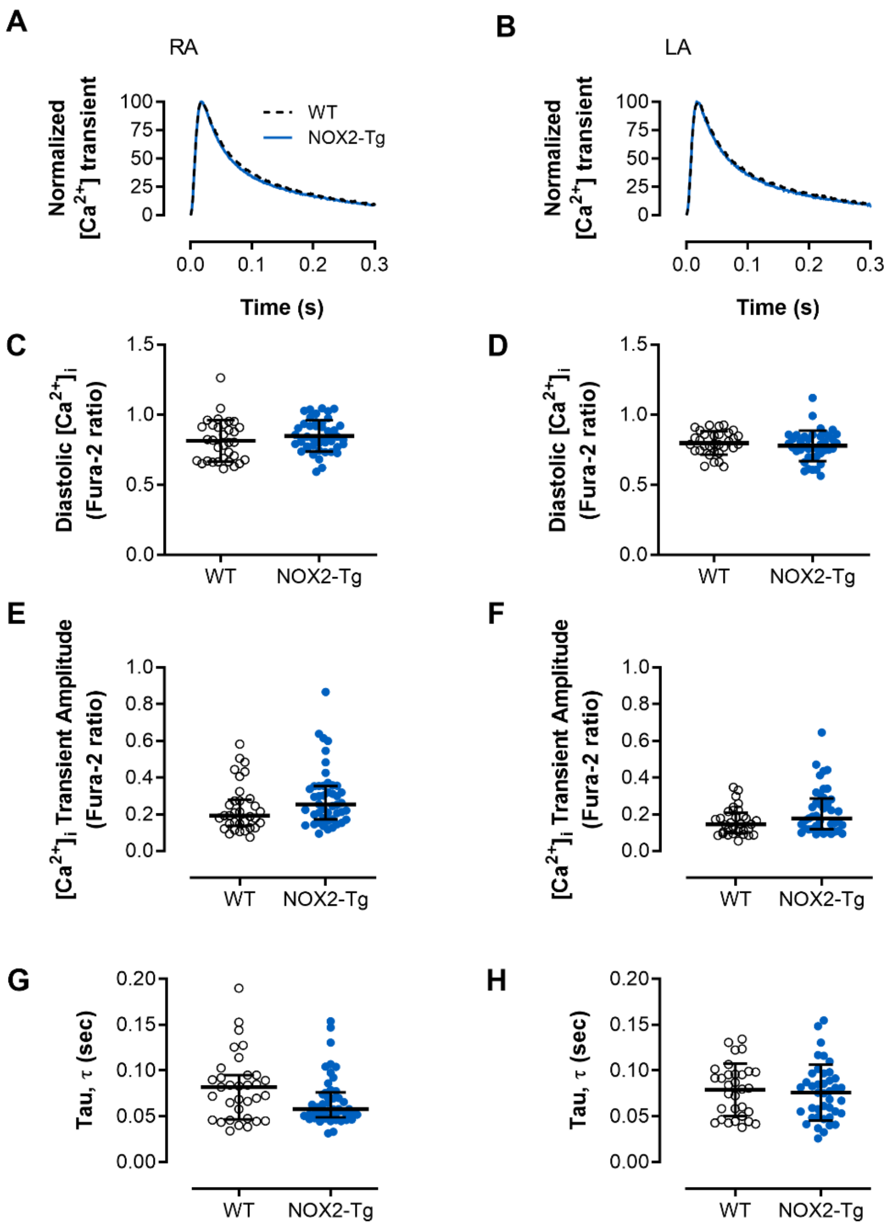

Supplementary Figure 3: Intracellular Ca^{2+} dynamics in ANG-II-stimulated NOX2-Tg and WT atrial myocytes. Overlays of averaged and normalized Ca^{2+} transients from RA (**A**) and LA (**B**) myocytes of WT and NOX2-Tg mice are shown. The diastolic Ca^{2+} content (**C-D**), amplitude of the Ca^{2+} transient (**E-F**), and rate of decay of the Ca^{2+} transient (tau, in sec) measured from the RA (n=33 and 46 cells from 9 mice per genotype) and LA (n=32 and 41 cells from 8 WT and 11 NOX2-Tg mice, respectively) were not significantly different between genotypes. Graphs show individual data points with medians and IQRs or means and SDs, as appropriate. Statistical significance was determined by two-tailed Student's t-test or Mann-Whitney *U* test using a hierarchical statistical clustering model.

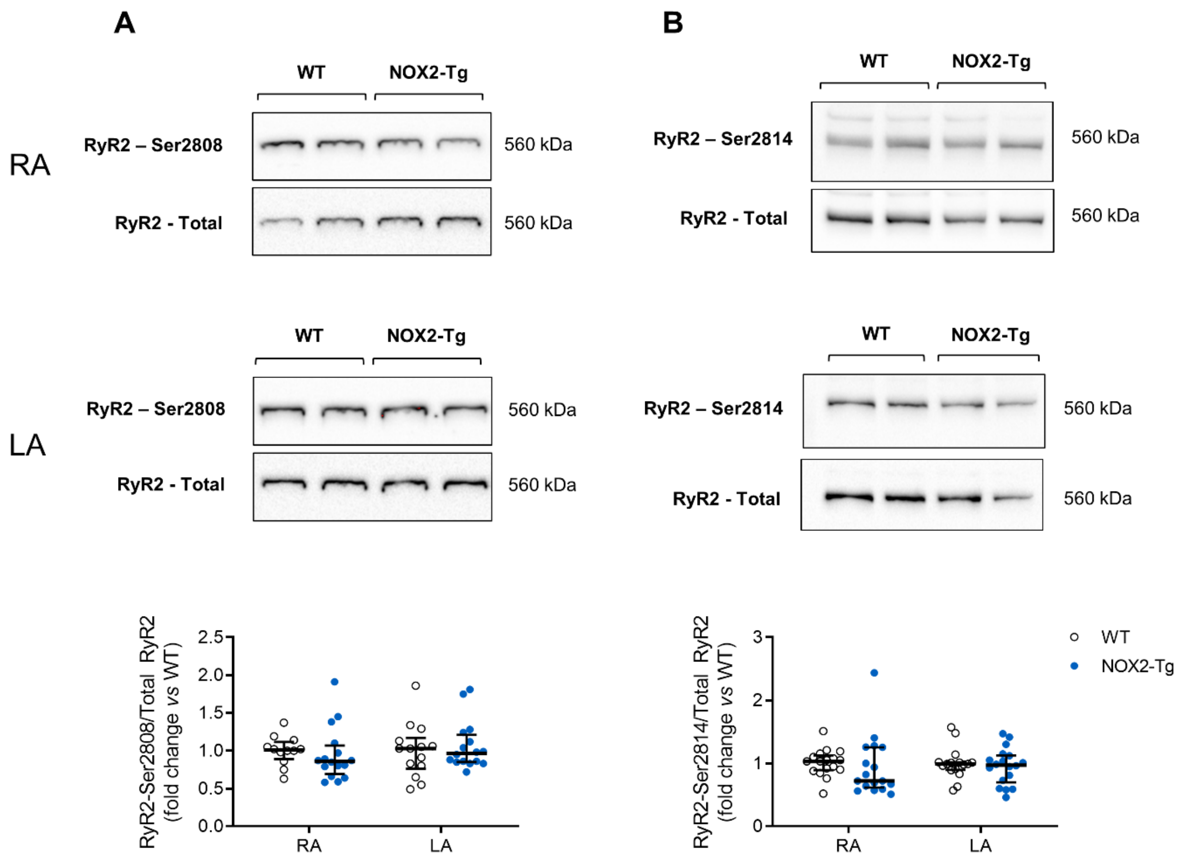

Supplementary Figure 4: RyR2 phosphorylation at Ser2808 and Ser2814 is unchanged in NOX2-Tg mice. (A) The RyR2 phosphorylated fraction at Ser2808 is similar between WT and NOX2-Tg mice both in the RA (n=12 and n=16 respectively, *P* >0.05) and in the LA (n= 14 and n=16, *P* >0.05). (**B**) Likewise, the RyR2 phosphorylated fraction at Ser2814 did not differ between genotypes (for the RA, n = 17 for both groups, *P* >0.05 and for the LA n=16 for WT and n=18 for NOX2-Tg, *P* >0.05). Top panels include representative Western blots from each atrium and genotype. Graphs show individual data points with medians and IQRs.

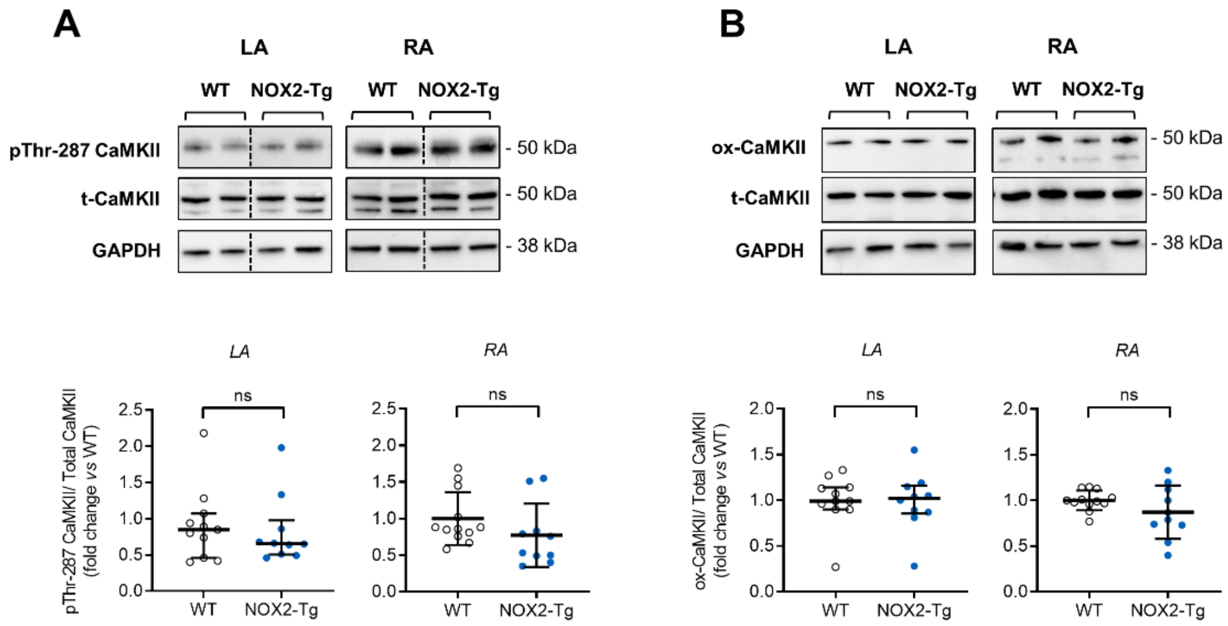

Supplementary Figure 5: Myocardial NOX2 overexpression does not affect phosphorylation or oxidation of CaMKII. (**A**) Phosphorylation of CaMKII on threonine-287 (pThr-287 CaMKII), assessed under reduced conditions, and total CaMKII (t-CaMKII) expression in LA and RA homogenates are not different between NOX2-Tg and WT mice (n=10-12 atria/genotype). (**B**) CaMKII oxidation (ox-CaMKII), assessed under non-reduced conditions, is not significantly different between NOX2-Tg and WT RA and LA tissue homogenates (n=10-12 atria/genotype). Un-paired Student's *t*-test or Mann-Whitney *U* test were used to determine statistical significance between groups and data are shown as individual data points with means and SDs or medians and IQRs, as appropriate. Dotted lines indicate where images, taken from different regions of the same western blot, were cropped.

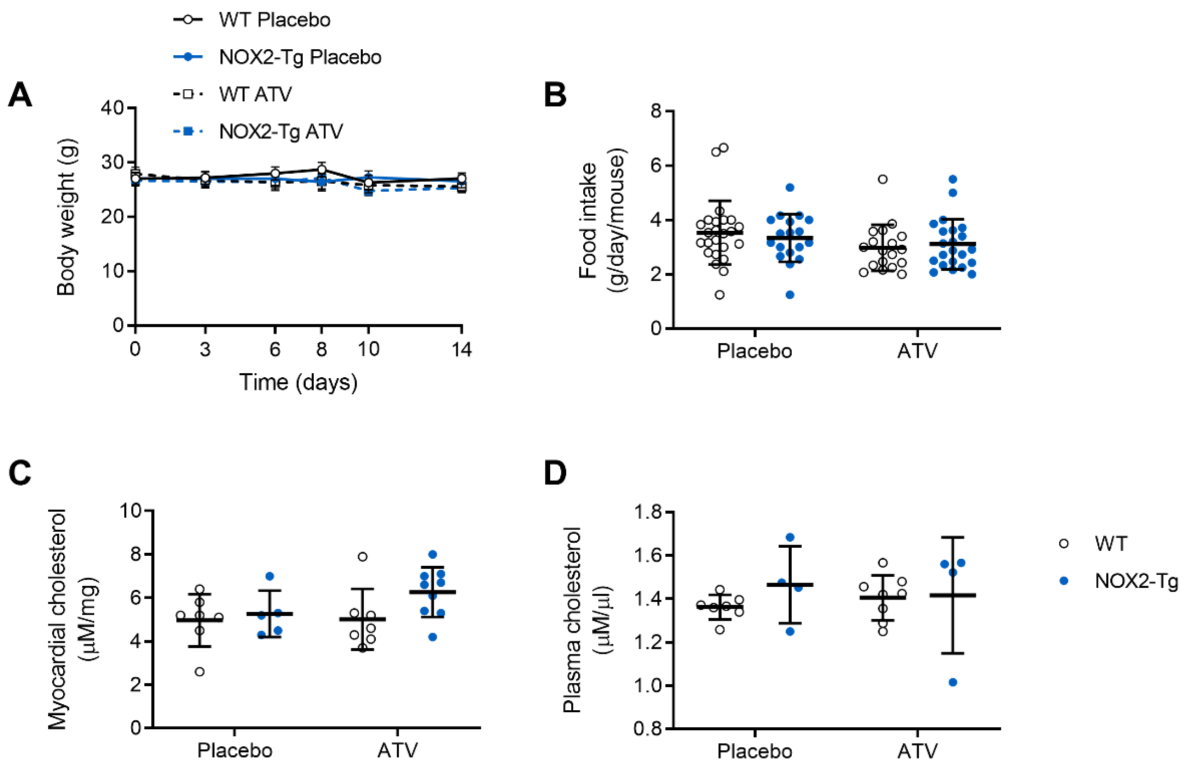

Supplementary Figure 6: Atorvastatin does not modify body weight, food intake, and myocardial or plasma cholesterol content. Comparison of body weight (**A**) and food consumption (**B**) between placebo- and ATV-treated WT and NOX2-Tg mice revealed no differences between groups. (**C**) There was no significant difference in the left ventricular free cholesterol content between WT and NOX2-Tg mice who received ATV (WT: n=7, 5.01 ± 1.4 µM/mg; NOX2-Tg: n=9, 6.27 ± 1.1 µM/mg) and those who received placebo (WT: n=7, 4.97 ± 1.2 µM/mg; NOX2-Tg: n=5, 5.26 ± 1.1 µM/mg). (**D**) Free cholesterol content in plasma samples was not affected by ATV treatment (WT: n=8, 1.41 ± 0.1 µM/µl; NOX2-Tg: n=4, 1.42 ± 0.27 µM/µl) compared with placebo (WT: n=7, 1.36 ± 0.06 µM/µl; NOX2-Tg: n=4, 1.47 ± 0.18). *P*-values were calculated by two-way ANOVA with Tukey *post-hoc* analysis. Graphs show individual data points with means and SDs.

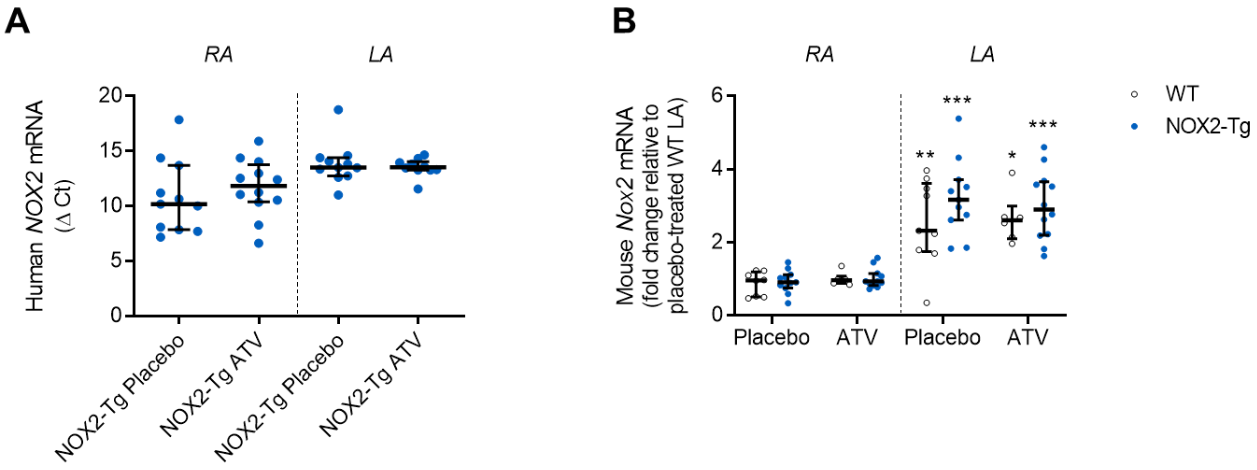

Supplementary Figure 7: Atrial NOX2 expression is unaffected by ATV treatment. (**A**) Expression of human *NOX2* mRNA in atrial tissue homogenates is similar between placebo- and ATV-treated NOX2-Tg mice (n=10-12/group). (**B**) Expression of murine *Nox2* mRNA in atrial tissue homogenates is similar between placebo- and ATV-treated WT and NOX2-Tg mice (n=6-12/group). One-way ANOVA with Tukey *post-hoc* analysis was used to determine statistical significance between groups. **P*<0.01, ***P*<0.001, and ****P*<0.0001 compared with respective RA. Graphs show individual data points with medians and IQRs.

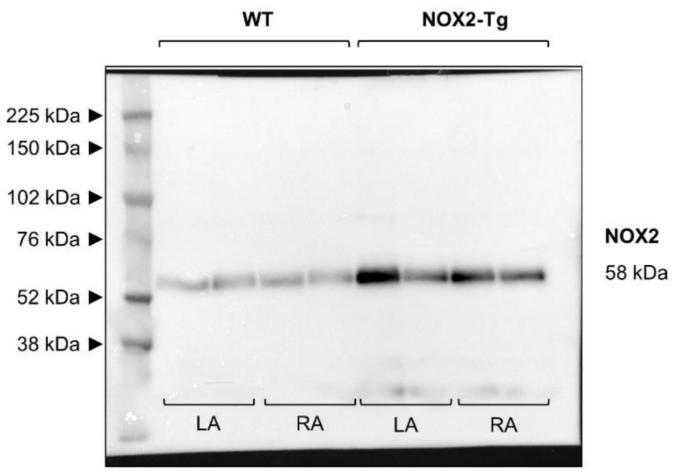

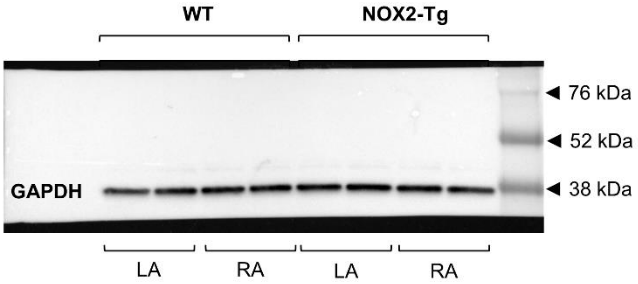

Supplementary Figure 8: Uncropped gels for western blots presented in Figure 1.

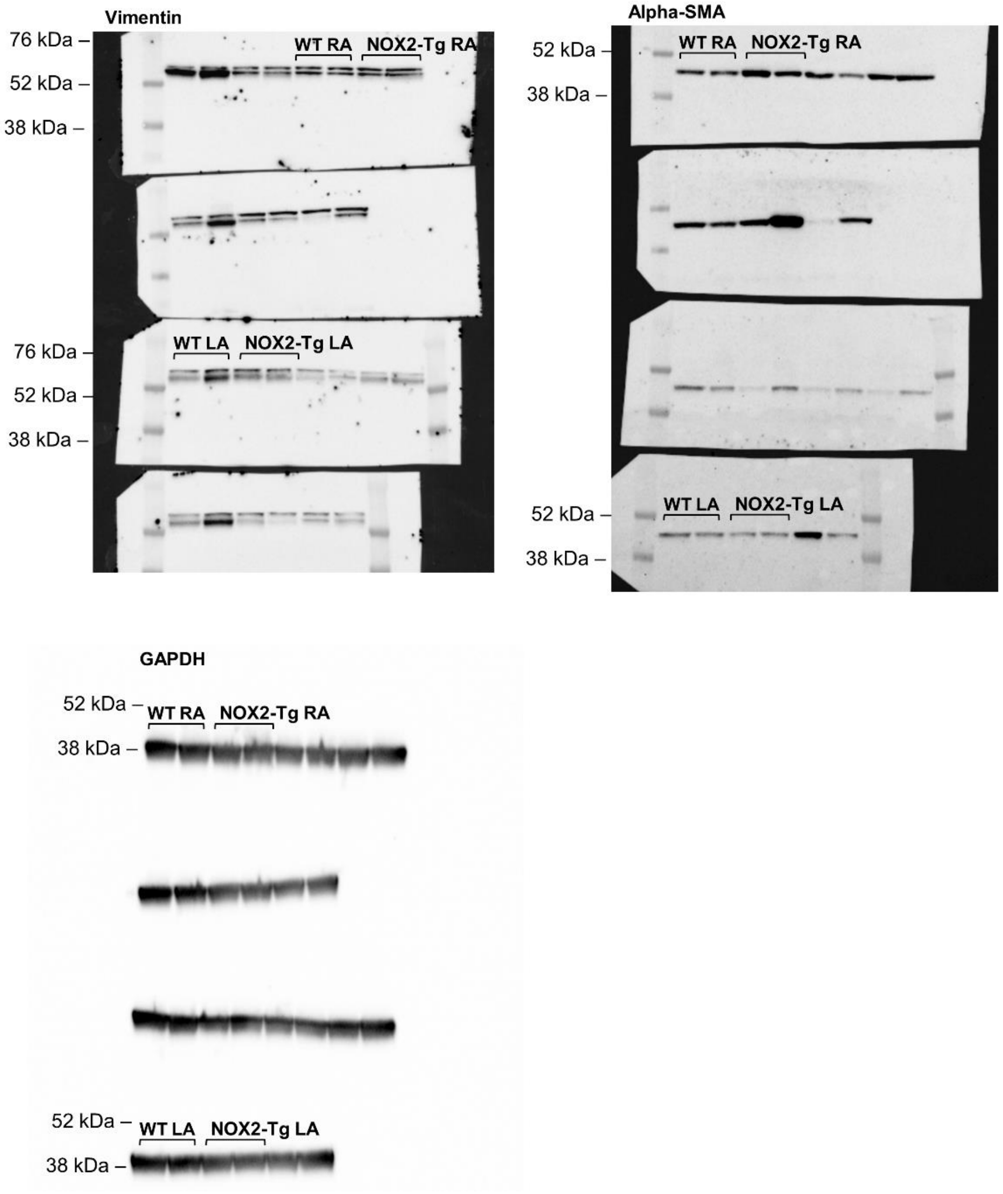

Supplementary Figure 9: Uncropped gels for western blots presented in Supplementary Figure 2A-B.

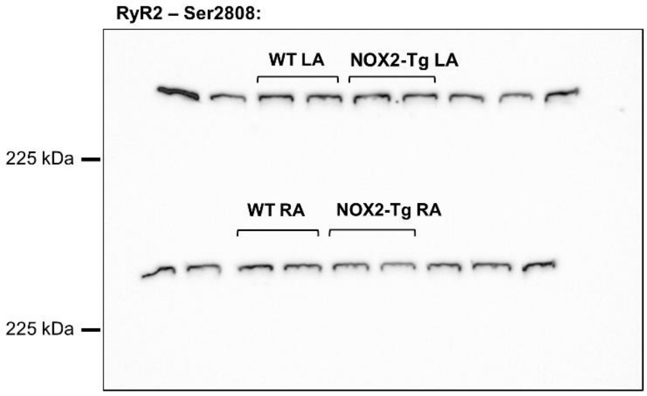

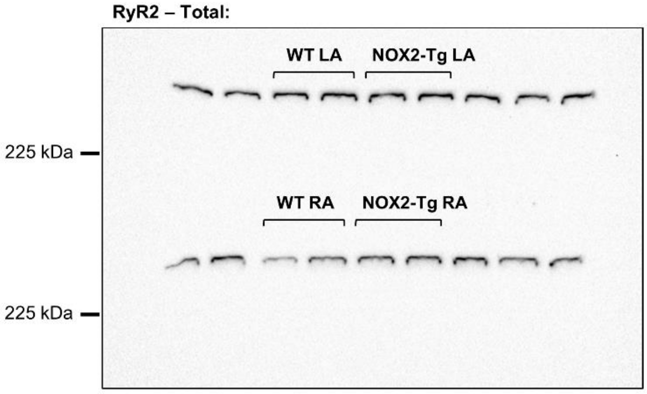

Supplementary Figure 10: Uncropped gels for western blots presented in Supplementary Figure 4A.

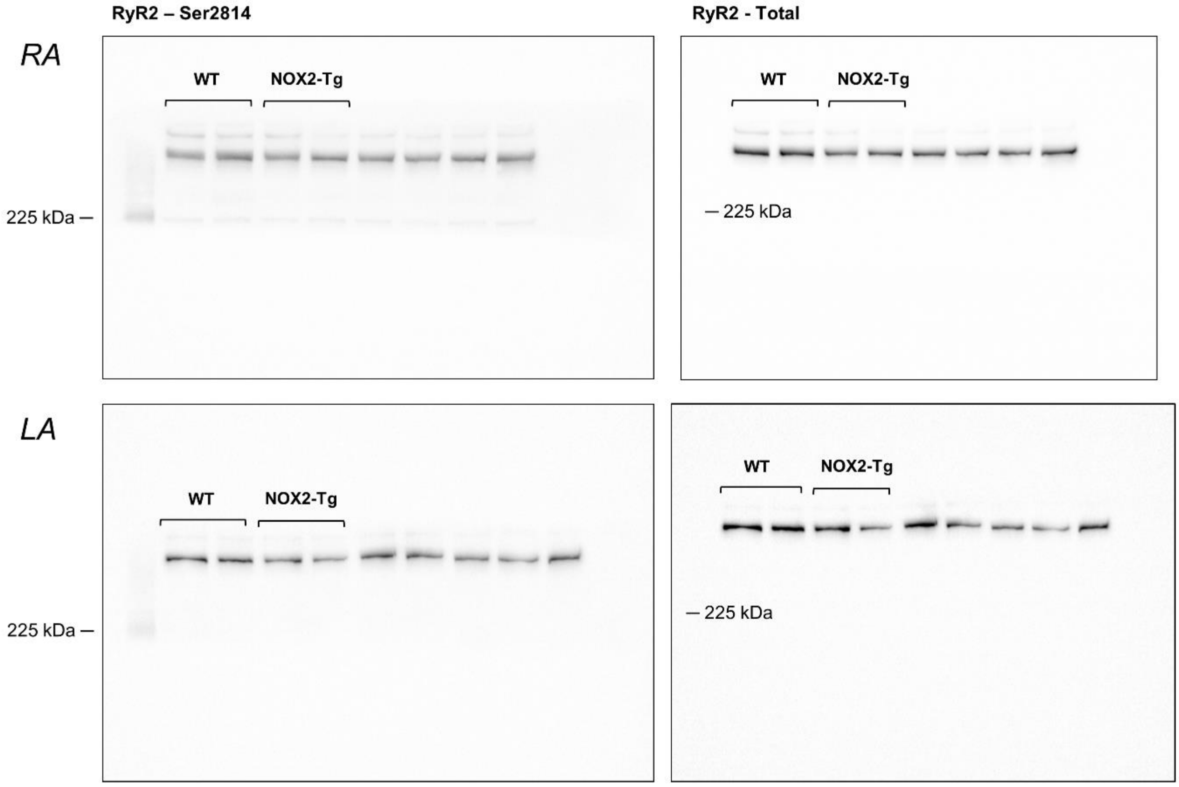

Supplementary Figure 11: Uncropped gels for western blots presented in Supplementary Figure 4B.

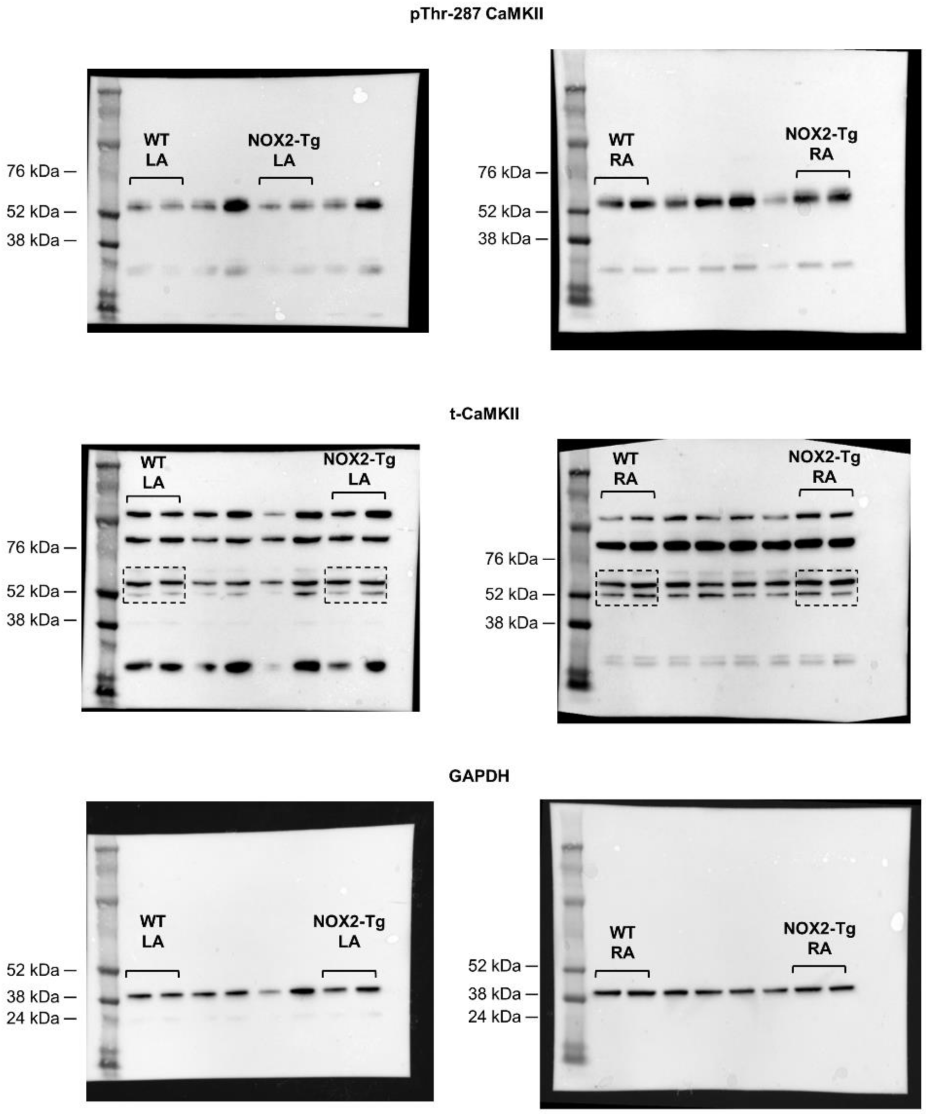

Supplementary Figure 12: Uncropped gels for western blots presented in Supplementary Figure 5A.

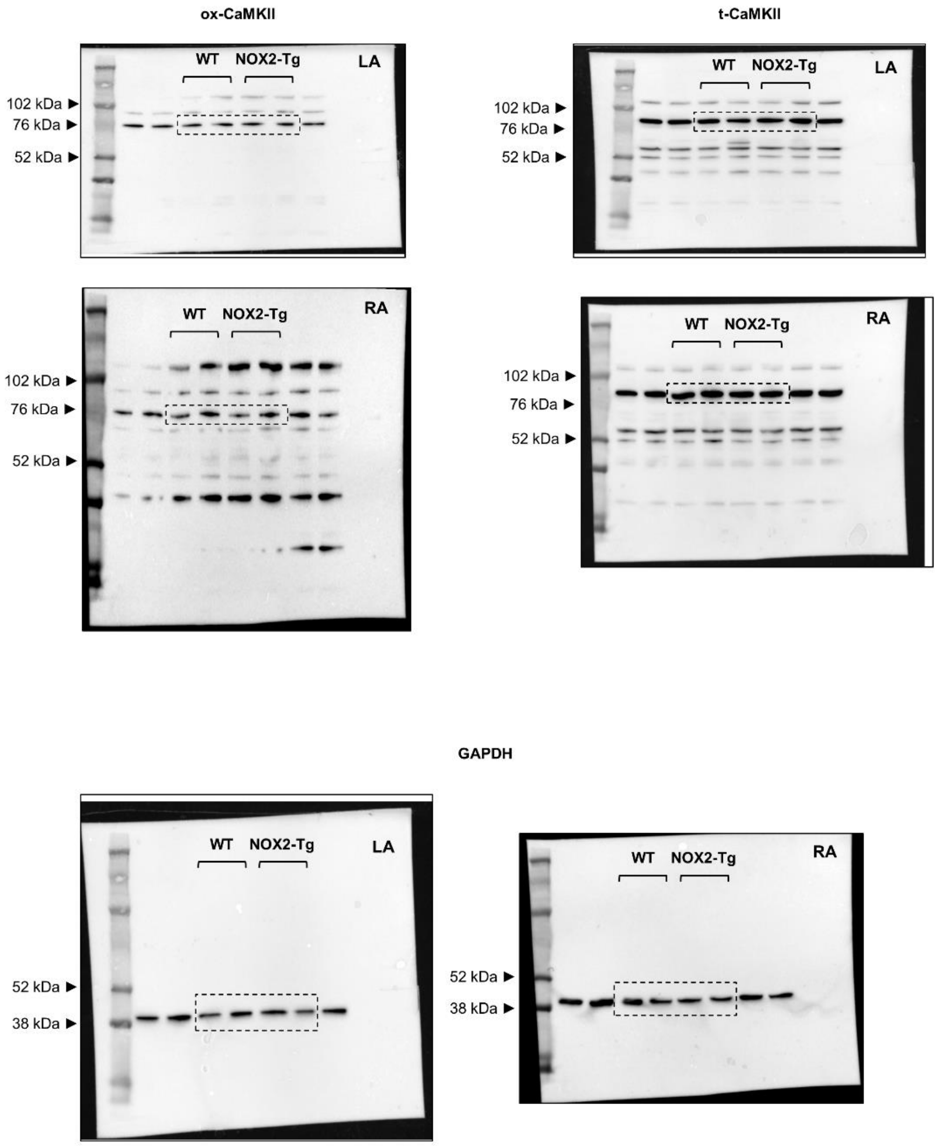

Supplementary Figure 13: Uncropped gels for western blots presented in Supplementary Figure 5B.

Supplementary Table 1: Summary of pacing-induced AF susceptibility and duration

	WT	NOX2-Tg	***P*-value**
Maximum duration (s)	47.1 (25 – 343)	63.7 (22.4 – 381)	0.58
**AF episodes ≥ 2 sec**			
Incidence (%, AF/Total)	69% (40/58)	88% (49/56)	0.037*
Probability (%)	9.3 (4.17 – 15.6)	11.5 (4.03 – 15.9)	0.83
Duration - mean (s)	19.7 (10.2 – 55.6)	30.5 (10.3 – 66.6)	0.46
Duration - cumulative (s)	148 (46.0 – 421)	143 (56.1 – 474)	0.67
**AF episodes ≥ 5 sec**			
Incidence (%, AF/Total)	65% (36/57)	80% (45/56)	0.06
Probability (%)	8.4 ± 0.84	9.0 ± 0.93	0.66
Duration – mean (s)	37.1 (16.1 – 78.1)	31 (15.8 - 106)	0.79
Duration – cumulative (s)	115 (50.1 – 414)	97.7 (49.9 – 570)	0.96
**AF episodes ≥ 10 sec**			
Incidence (%, AF/Total)	61% (35/57)	77% (43/56)	0.10
Probability (%)	5.9 (2.56 – 8.51)	4.7 (2.56 – 8.97)	0.95
Duration – mean (s)	31.8 (18.7 – 111)	36.6 (20.1 – 253)	0.40
Duration – cumulative (s)	118 (39.2 – 411)	69 (40.2 – 584)	0.81

Comparison of AF incidence, probability, and duration between WT and NOX2-Tg mice. Data shown are mean ± SD or median (25th-75th percentile). *P*-values comparing continuous data were calculated by a two-tailed unpaired Student's t-test or Mann-Whitney *U* test, as appropriate. Categorical values were analysed using Fisher's exact test.
*P<0.05

Supplementary Table 2: Summary of APD measurements in paced WT and NOX2-Tg atria

	WT		NOX2-Tg	
	**Baseline**	**ANGII**	**Baseline**	**ANGII**
**RA**				
APD30	5.03 ± 0.44	5.95 ± 1.25	5.15 ± 0.50	5.68 ± 1.09
APD50	6.71 ± 0.63	8.21 ± 1.77	6.92 ± 0.78	8.15 ± 1.87
APD80	10.64 ± 1.04	13.89 ± 3.63	10.73 ± 1.44	13.10 ± 2.71
**LA**				
APD30	4.88 ± 0.34	5.90 ± 1.35	5.11 ± 0.35	5.39 ± 0.62
APD50	6.44 ± 0.34	8.08 ± 2.07	6.97 ± 0.42	7.47 ± 0.85
APD80	10.1 ± 0.68	13.36 ± 2.55	10.9 ± 0.89	14.51 ± 3.83

Summarized data APD30, APD30, and APD80 measured from RA and LA tissues paced at a cycle length of 100-ms at baseline and following incubation with ANG-II. Data shown as mean ± SD and statistical significance was determined using unpaired Student’s t-test. n= 11 and 9-10 for WT and NOX2-Tg atria, respectively.

Supplementary Table 3: Summary of pacing-induced AF susceptibility and duration between placebo and ATV-treated mice

	**PLACEBO**		**ATV**	
	**WT**	**NOX2-Tg**	**WT**	**NOX2-Tg**
Maximum duration (s)	67.5 (31.7 – 433)	117 (40.4 – 381)	43.2 (25.4 – 242)	64.6 (22.3 – 261)
**AF episodes ≥ 2s**				
Incidence (%, AF/Total)	76% (19/25)	93% (25/27)	81% (22/27)	84% (27/32)
Probability (%)	12.2 ± 6.0	13.0 ± 7.83	14.1 ± 6.18	10.7 ± 5.37
Duration – mean (s)	25.5 (10.9 – 62.3)	31 (16.6 – 78.3)	14 (10.3 – 45)	18.5 (10.9 – 68.8)
Duration – cumulative (s)	208 (74 – 520)	246 (116 – 474)	**139 (44 – 309)***	**129 (53 – 418)***
**AF episodes ≥ 5s**				
Incidence (%, AF/Total)	76% (19/25)	93% (25/27)	78% (21/27)	84% (27/32)
Probability (%)	8.58 ± 4.85	10.7 ± 5.92	10.3 ± 4.34	7.34 ± 4.03
Duration – mean (s)	49.6 (29.7 – 103)	31 (17.4 – 96.4)	19.9 (12.9 – 57.8)	24.8 (16.5 – 96.4)
Duration – cumulative (s)	201 (83.8 – 508)	207 (103 – 446)	110 (77.4 – 340)	124 (49.7 – 411)

Comparison of AF incidence, probability, and duration between placebo and ATV-treated WT and NOX2-Tg mice. Data shown are mean ± SD or median (25th-75th percentiles). P-values comparing continuous data were calculated with two-way ANOVA and Tukey multiple comparison test. Categorical variables were analysed with Fisher's exact test. *P=0.038 *vs* placebo

Supplementary Table 4: Echocardiographic measurements and ECG parameters in placebo and ATV-treated mice

	PLACEBO		ATV	
	**WT**	**NOX2-Tg**	**WT**	**NOX2-Tg**
**Echocardiography**	*n* = 6	*n* = 6	*n* = 5	*n* = 7
LVPWd (mm)	0.84 ± 0.06	0.92 ± 0.19	0.89 ± 0.12	0.89 ± 0.17
LVPWs (mm)	1.36 ± 0.20	1.33 ± 0.15	1.33 ± 0.24	1.38 ± 0.24
LVIDd (mm)	3.41 ± 0.39	3.61 ± 0.23	3.51 ± 0.52	3.69 ± 0.55
LVIDs (mm)	2.0 ± 0.45	2.2 ± 0.27	2.15 ± 0.43	2.27 ± 0.57
LV mass (mg)	80.9 ± 20.7	94.5 ± 24.1	92.4 ± 43.6	95.3 ± 30.6
Indexed LV mass (mg/g)	3.78 ± 0.90	3.86 ± 1.06	4.13 ± 1.16	4.35 ± 0.70
EF (%)	73.1 ± 9.72	70.3 ± 5.52	69.9 ± 6.71	69.6 ± 9.91
FS (%)	42.0 ± 9.12	39.2 ± 4.7	38.8 ± 5.71	39.2 ± 7.25
Mitral valve E/A ratio	1.82 ± 0.17	1.75 ± 0.20	1.92 ± 0.58	1.89 ± 0.38
Combined E/E’ ratio	28.9 ± 7.43	27.8 ± 5.03	34.6 ± 1.37	36.5 ± 12.2
**ECG parameters**	*n* = 29	*n* = 30	*n* = 32	*n* = 33
RR (ms)	130 ± 19	138 ± 18	134 ± 19	135 ± 18
P wave (ms)	22.6 ± 1.66	22.4 ± 2.13	22.5 ± 1.42	22.2 ± 2.0
PQ (ms)	41.5 ± 3.13	40.9 ± 4.61	42.1 ± 3.83	40.9 ± 3.0
QRS (ms)	8.09 ± 0.80	7.58 ± 0.87	57.4 ± 7.46	60.7 ± 7.30
QTc (ms)	51 ± 4.21	54.0 ± 4.79	49.6 ± 5.28	52.4 ± 4.93

Surface ECG parameters and echocardiographic measurements evaluating left ventricular mass, and systolic and diastolic function of placebo and ATV-treated WT and NOX2-Tg mice. Data shown are mean ± SD. Data were analysed by two-way ANOVA with Tukey multiple comparison test. *N* – number of mice; LVPWd and LVPWs – left ventricular posterior wall thickness at end-diastole and end-systole; LVIDd and LVIDs – left ventricular internal diameter at end-diastole and end-systole; EF – ejection fraction; FS – fractional shortening; E – early mitral valve inflow velocity (passive filling); A – late mitral valve inflow velocity (active filling); E’ – early tissue Doppler mitral valve annulus velocity.

Supplementary Table 5. Materials List

	Source	Identifier
Antibodies		
anti-rabbit RyR2 (Phospho-Ser2814)	Badrilla Ltd.	A010-31
anti-rabbit RyR2 (Phospho-Ser2808)	Badrilla Ltd.	A010-30AP
anti-mouse RyR2	ThermoFisher Scientific	MA3-925
anti-mouse $gp91^{phox}$	BD Transduction Lab.	611414
Monoclonal anti-GAPDH peroxidase	Sigma Aldrich	G9295
Total CaMKII	Cell Signaling Technology	3362
anti-rabbit α-smooth muscle actin	Cell Signaling Technology	19245S
anti-mouse vimentin	Exbio Antibodies	EXB-11-460
Chemicals		
Angiotensin II	Sigma Aldrich	A9525
Blebbistatin	RD Systems	1760
Caffeine	Sigma Aldrich	C0750
di-4-ANEPPS	Sigma Aldrich	D8064
Fura-2 AM	ThermoFisher Scientific	F1221
NADPH	Sigma Aldrich	N5130
Pluronic F-127, 20% solution in DMSO	Sigma Aldrich	59004
Pierce protease inhibitor mini tablets	ThermoFisher Scientific	88665
cOmplete mini protease inhibitor tablets	Sigma Aldrich	11836153001
RIPA (10X)	Sigma Aldrich	20-188
TaqMan		
Tetracaine	Sigma Aldrich	T7383
Tiron	Sigma Aldrich	172553
Commercial Assays		
Amplex Red Cholesterol Assay Kit	ThermoFisher Scientific	A12216
BCA Protein Assay Kit	ThermoFisher Scientific	23225
mirVana miRNA Isolation Kit	ThermoFisher Scientific	AM1561
QuantiTect Reverse Transcription Kit	Qiagen	205313
RNeasy Mini Kit	Qiagen	74104
TaqMan Gene Expression Master Mix	ThermoFisher Scientific	4369016
